# Achieving High‐Instantaneous Pulse Power in Contact‐Separation TENG via Series Capacitive Voltage Control Architecture

**DOI:** 10.1002/advs.77535

**Published:** 2026-09-10

**Authors:** Ajay Haridas CP, Prosenjit Das, Kinsuk Naskar, Titash Mondal

**Affiliations:** ^1^ Rubber Technology Centre Indian Institute of Technology Kharagpur India; ^2^ Department of Electrical Engineering Indian Institute of Technology Kharagpur India

**Keywords:** capacitive coupling architecture, contact‐separation mode, high‐power energy harvesting, self‐powered systems, triboelectric nanogenerator

## Abstract

Contact‐separation triboelectric nanogenerators generate high voltages but deliver minimal power due to impedance mismatch and premature charge dissipation. Here, we introduce a capacitive charge reservoir architecture that decouples energy accumulation from discharge triggering, enabling sequential voltage amplification through floating‐layer capacitive coupling. The device employs intermediate charge reservoirs positioned between the triboelectric assembly and spark‐gap switch, which accumulate charge over the contact‐separation cycle before threshold‐triggered discharge. This architecture, namely, Series Capacitive Voltage Control Architecture TENG (SCVC‐TENG), achieves a power density of 7.3 MW/m^2^ and current density of 5.36 kA/m^2^ at optimal load, representing a 5.93 × 10^5^ and 2.55 × 10^5^ times improvement over conventional contact‐separation design. The charge reservoir mechanism enables controlled energy release, leading to a pulsed, high‐power mode of operation for 221 W commercial lamps or a continuous mode for powering low‐power electronics through inductor‐based energy management. The device maintains 99.3% performance over 11 500 cycles during continuous charging, demonstrating that architectural decoupling of charge accumulation from discharge dynamics provides a pathway to high‐instantaneous power, along with continuous contact‐separation energy harvesting without sacrificing mechanical simplicity or durability.

## Graphical abstract

Conventional contact‐separation triboelectric nanogenerators generate high voltage but deliver limited power due to premature charge dissipation. Here, a capacitive charge reservoir architecture decouples charge accumulation from discharge triggering, enabling sequential voltage amplification via floating‐layer capacitive coupling. The resulting SCVC‐TENG achieves 7.3 MW/m^2^ power density and 5.36 kA/m^2^ current density, a 593 000 and 255 000‐fold enhancement, respectively, over conventional design and is capable of powering 221 W combinedly rated lamps while retaining 99.3% output over 11 500 cycles, without sacrificing mechanical simplicity.

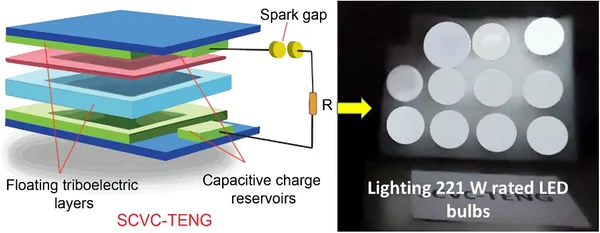

## 1 Introduction

The rapid advancement of Internet of Things (IoT) devices, sensors, and wearable electronics has necessitated a radical shift from traditional batteries to sustainable energy harvesters, driven by limited lifespans, heavy packaging, and environmental concerns [1]. This driven need for sustainable alternatives has popularised TENGs that can harvest unlimited waste kinetic energy from the ambient environment [2]. Numerous TENG designs have been developed to efficiently convert wasted mechanical energy from sources such as wind [3, 4], human body movements [5], tidal waves [6, 7], and raindrops [8] into electrical energy. Since their introduction in 2012, TENGs have demonstrated advantages in ease of fabrication, material diversity, lightweight construction, and mechanical flexibility [9, 10, 11]. In principle, TENGs work on the coupled mechanisms of the triboelectric effect and electrostatic induction [12, 13]. Among the four primary TENG operating modes, i.e., contact‐separation, lateral sliding, single‐electrode, and freestanding, the contact‐separation mode has garnered significant attention due to its simplicity in fabrication, ease of operation, and high voltage generation capability [14]. However, conventional contact‐separation TENGs (CCS TENGs) face a fundamental limitation: despite generating kilovolt‐level voltages, their power output remains orders of magnitude lower than sliding‐mode devices [15].

Recent efforts to enhance TENG power output have pursued several strategies, including surface modification, novel material pairings, structural optimization, and hybrid energy‐harvesting systems, to increase charge density and energy conversion efficiency [1, 10, 16, 17, 18, 19, 20, 21]. Material‐based approaches have focused on incorporating conductive fillers or engineering microstructural features to enhance charge‐transfer efficiency [22]. Structural innovations have proven particularly effective to enhance the charge and power output of the TENG [23, 24]. In a recent study, an opposite charge enhanced‐transistor‐like design TENG achieved an ultrahigh instantaneous power density of over 10 MW/m^2^, and commercial lamps rated up to 180 W were lighted with an effective area of 25 cm^2^ [25]. However, such high‐performance devices predominantly operate in sliding mode, where continuous friction between moving surfaces generates substantial charge but causes accelerated wear of triboelectric layers [26]. Maintaining performance in sliding‐mode TENGs requires either frequent replacement of worn surfaces or introduction of lubricants and low‐friction materials, increasing system complexity and cost [27, 28].

In contrast, CCS TENGs experience minimal mechanical wear during operation, as surfaces engage and disengage without lateral motion. This characteristic extends device lifetime and eliminates the need for friction management, making contact‐separation devices more suitable for long‐term deployment in applications such as wearable energy harvesters, self‐powered sensors, and vibration energy recovery systems [29, 30, 31]. The operational simplicity and durability of the contact‐separation mode create a compelling case for improving its power output rather than accepting the maintenance burden of sliding‐mode alternatives. The fundamental limitation of CCS TENGs lies in the dynamics of charge accumulation and release. During contact‐separation motion, charge is generated incrementally across capacitive interfaces as triboelectric layers engage and disengage. In conventional designs, any discharge mechanism (such as a spark gap, external load, or diode rectifier) acts directly on the triboelectric assembly. This creates a temporal bottleneck as the charge begins to dissipate before the contact‐separation cycle is fully completed, preventing the maximum voltage build‐up. The result is premature discharge at sub‐optimal voltage and current levels.

We address this limitation through a series capacitive voltage control (SCVC) mechanism aided by a capacitive charge reservoir (CCR) architecture, which decouples charge accumulation dynamics from discharge triggering and floating layers, which maintains the high potential in the system. The innovation centers on intermediate charge storage nodes, i.e., capacitive air gaps positioned between the triboelectric floating assembly and the discharge control element (spark gap switch). These reservoirs, in conjunction with the spark gap switch, perform three critical functions: (1) accumulate charge incrementally throughout the contact‐separation cycle without immediate dissipation, (2) buffer the triboelectric assembly from premature discharge, and (3) enable threshold‐triggered energy release only when accumulated voltage reaches optimal levels for power delivery. Thus, this enhanced TENG achieves a high‐power output by series capacitive voltage control, hence termed as Series Capacitive Voltage Control Architecture TENG (SCVC‐TENG).

Our SCVC‐TENG implements this concept through a layered architecture comprising floating triboelectric layers (graphene‐enhanced TPU composite and PTFE), a floating electrode layer, a static electrode layer, and dual capacitive charge reservoirs (air gap capacitances) that feed into an automatic spark‐gap switch. The charge reservoirs allow the system to build voltage sequentially across multiple capacitive stages during contact, analogous to a multi‐stage charge pump. When the accumulated voltage exceeds the spark gap breakdown threshold during separation, rapid discharge delivers the stored energy to the load. A systematic comparison with control architectures (Figure 3) demonstrates that integrating a charge reservoir and a floating structure is essential for performance enhancement, and the complete SCVC architecture with CCRs, floating layers, and spark gaps achieves 2.55 × 10^5^‐fold current enhancement and 5.93 × 10^5^‐fold power improvement compared to CCS‐TENG, i.e., a basic architecture without capacitive charge reservoirs, floating electrode layer, or spark gap, used throughout this work as the baseline architecture for direct performance comparison. Combined with inductor‐based energy management, the device successfully operates 221 W combined‐rated commercial LED lamps in pulsed mode and continuously powers low‐energy electronics at 4 Hz. Performance stability over 11 500 cycles confirms mechanical robustness. These results establish that decoupling charge accumulation from discharge dynamics via a capacitive reservoir architecture enables CS TENGs to achieve power densities approaching those of sliding‐mode devices while retaining operational simplicity and long‐term durability.

## 2 Power Enhancement via Floating Triboelectric Layers with Charge Reservoir Architecture in Combination with Spark Gap and its Operating Principle

**FIGURE 1:**
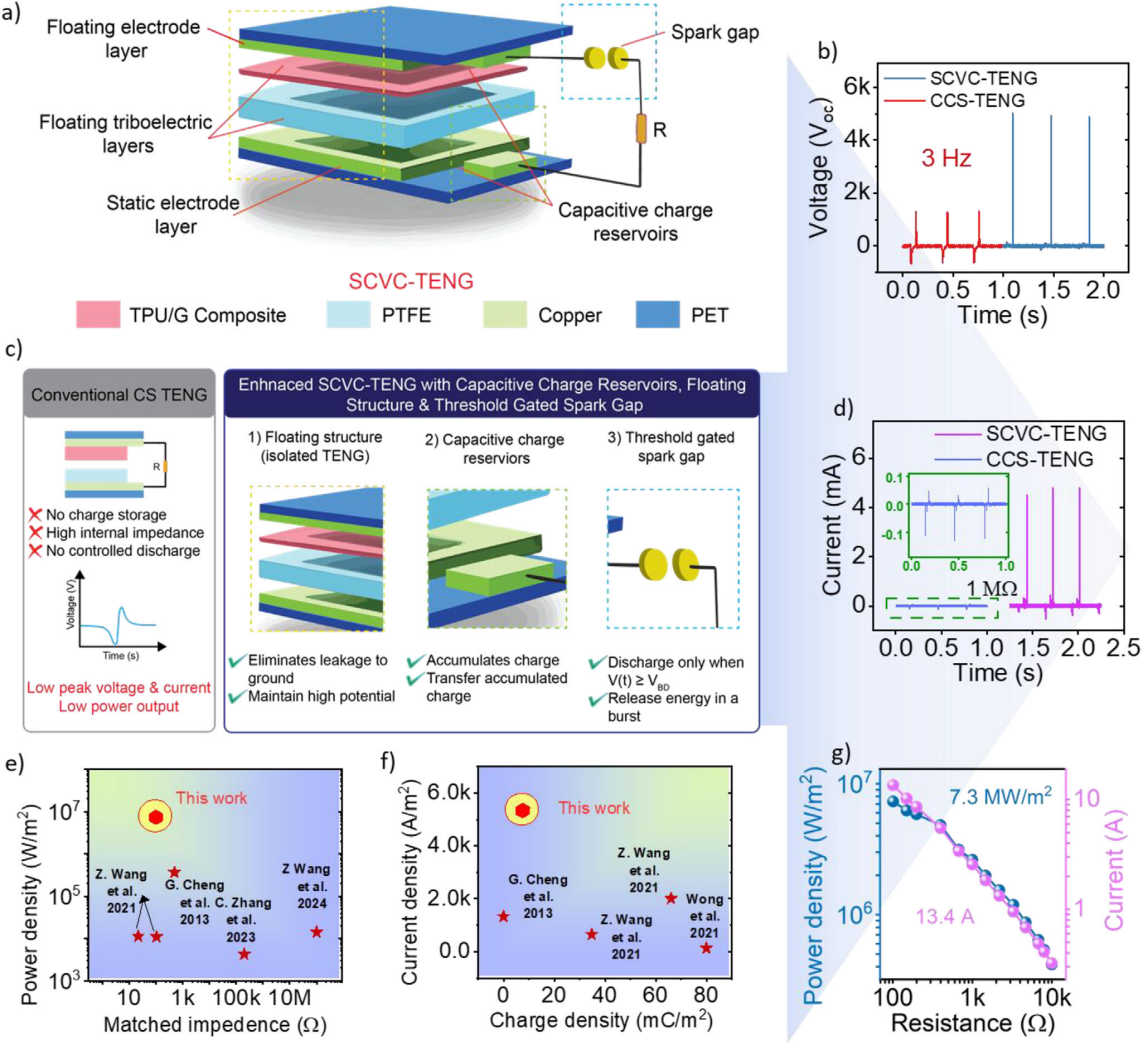
(a) Schematic illustration of Sequential Capacitive Voltage‐Coupled TENG (SCVC‐TENG). (b) Output voltage comparison of SCVC‐TENG and CCS‐TENG at 3 Hz working frequency. (c) Comparison of CCS‐TENG performance contributions and the enhanced SCVC‐TENG with contributions of each component in the modified architecture. (d) Output current comparison of SCVC‐TENG and CCS‐TENG on a 1 MΩ load. (e) Comparison of the instantaneous power density and the matched impedance obtained in this work with other contact‐separation mode TENG reports (the background gradient serves as an indicator of performance, with optimal values concentrated in the upper left region of the plot). (f) Comparison of the instantaneous current density and the charge density obtained in this work with other contact‐separation mode TENG reports (the background gradient serves as an indicator of performance, with optimal values concentrated in the upper right region of the plot). (g) Power density and current at low load resistances from 100 to 10 kΩ load.

The SCVC‐TENG architecture (Figure 1a) comprises five functional elements working synergistically to achieve high‐power output: two floating triboelectric layers (FTL‐TPU/G and FTL‐PTFE), one floating electrode layer (FEL), one static electrode layer (SEL), and two capacitive charge reservoirs (CCR‐top and CCR‐bottom). The innovation lies in integrating a floating layered configuration for voltage multiplication with intermediate charge reservoirs for temporal energy accumulation, controlled by threshold‐gated spark discharge, as illustrated in Figure 1b.

**FIGURE 2:**
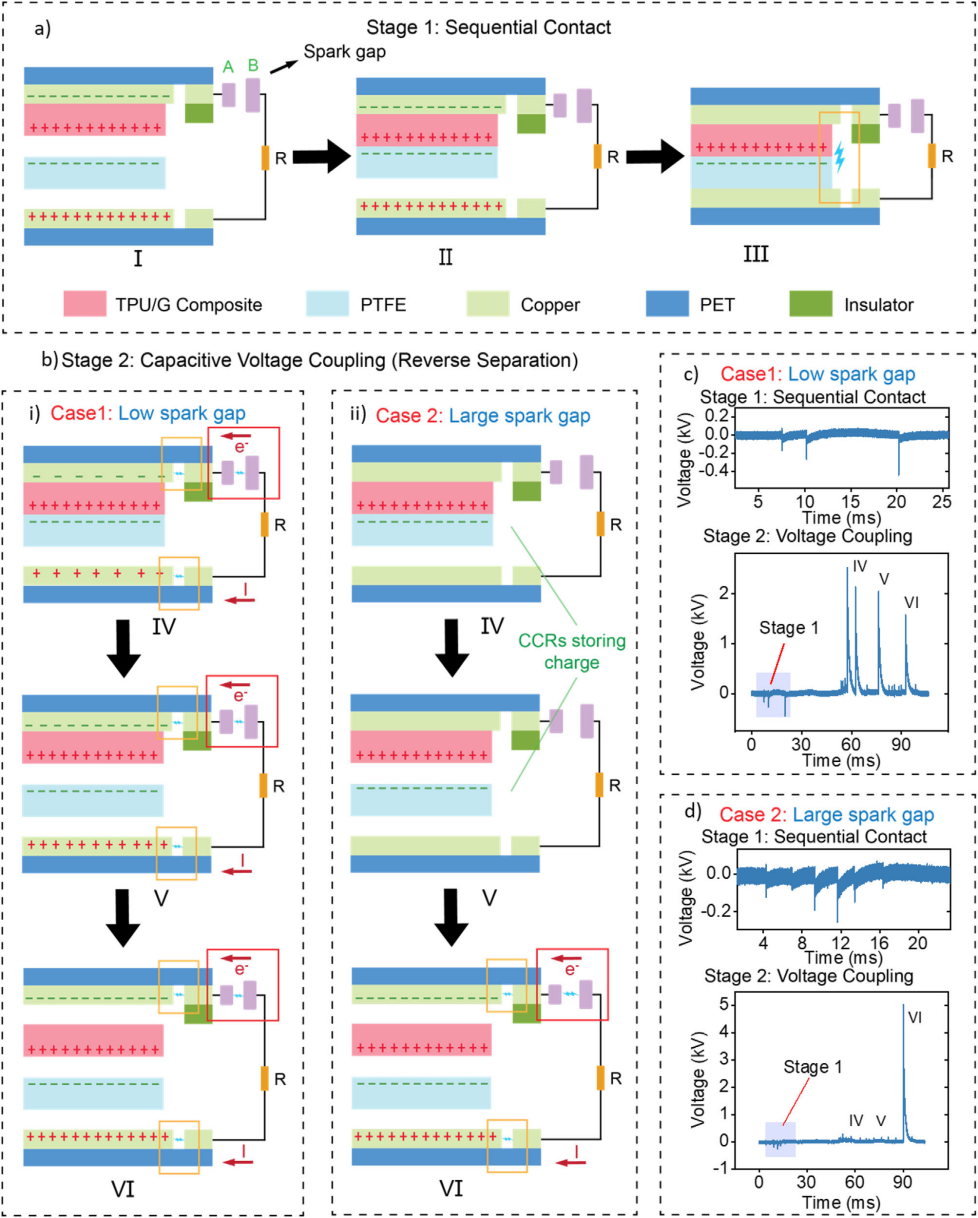
Schematic diagram of the working principle of the SCVC‐TENG. (a) Illustration of various steps of sequential contact (stage 1). (b) Illustration of reverse separation of contacted layers and their charge flow during (i) low spark gap and (ii) large spark gap. (c) The generated voltage during one working cycle of the TENG, when the spark gap is low. (d) The generated voltage during one working cycle of the TENG, when the spark gap is large.

During operation, the system functions as a series network of dielectric capacitors and time‐varying air gap capacitors, as illustrated in Figure S1. However, the effective capacitance of the system will be mainly contributed by the air‐gap capacitors, as described in Note S1. Thus, the effective capacitance (*C_eff_ (t)*) is given by:

(1) $$\frac{1}{C_{eff}\left(t\right)}=\frac{1}{C_{\mathrm{FEL}-\mathrm{TPU}}\;}+\frac{1}{C_{\mathrm{TPU}-\mathrm{PTFE}}\;}+\frac{1}{C_{\mathrm{PTFE}-\mathrm{SEL}}}$$

where each capacitance is determined by the air‐gap distance and the contact area at the respective interface. During contact, triboelectric charging creates surface charge densities +σ on TPU/G and ‐σ on PTFE. These bound charges induce free charges on the FEL and SEL through electrostatic induction. Crucially, because the FEL is floating and the CCRs are placed between the triboelectric assembly and the spark gap, the charge cannot escape. It remains trapped until full contact occurs and the distance between FEL and SEL falls below the breakdown voltage of the generated potential. At this point, spark discharge occurs between the SEL and FEL as illustrated in Figure 2a(III) to neutralize the whole triboelectric system.

As separation begins, the capacitances decrease while charge Q remains temporarily trapped due to the presence of CCRs. From the fundamental capacitor relation Q = CV, if C decreases while Q is conserved, voltage V must increase proportionally:

(2) $$V_{\mathrm{separation}}=Q/C_{\mathrm{separation}}\;>>\;V_{\mathrm{contact}}=Q/C_{\mathrm{contact}}$$

Because the FEL is floating, and direct discharge of charge is blocked by CCRs, this voltage enhancement occurs independently at each capacitive interface, and these voltages add in series:

(3) $$V_{\mathrm{total}}=V_{\mathrm{FEL}-\mathrm{TPU}}+V_{\mathrm{TPU}-\mathrm{PTFE}}+V_{\mathrm{PTFE}-\mathrm{SEL}}$$

This is the essence of floating electrode voltage multiplication as individual contributions sum rather than being shorted to ground.

The critical innovation lies in the positioning of capacitive charge reservoirs (Figure 1a,c) between the triboelectric assembly and the discharge trigger (spark gap). In conventional TENG designs, output electrodes connect directly to the load or discharge mechanism, causing charge dissipation to begin immediately when voltage exceeds the discharge threshold and preventing full charge accumulation during the contact‐separation cycle (Figure 1c). The CCR architecture breaks this direct coupling by treating the air gaps as capacitive energy reservoirs that store charge incrementally during contact‐separation without triggering premature discharge (Figure 2). This decoupling enables the system to accumulate charge till breakdown potential is reached, then release it through threshold‐triggered spark discharge, which causes the temporal energy compression that delivers instantaneous power orders of magnitude higher than average charging power. In grounded configurations, charge flows to ground during separation, dissipating energy continuously, whereas in the floating configuration with charge reservoirs, charge redistribution occurs internally within the closed capacitive network until the spark gap breakdown threshold is reached, enabling controlled high‐power release. This device is analogous to a multi‐stage capacitive voltage booster, where voltage is built up stage by stage by stacking charge movement across multiple capacitors in sequence. The dielectric gaps between each layer and the spark gap are analogous to “capacitors between booster stages”. The FEL and SEL can be regarded as “intermediate charge storage plates,” and the sequential contact‐and‐separation mechanism as the “clock/pulse signal” driving the boost. The induced charge in SCVC‐TENG is redistributed during the sequential contact‐and‐separation mechanism, as in charge transfer between capacitors during the boosting stage, where diodes control the flow of charges; here, the CCRs (Figure S1a) control the flow of charges. The final enhanced output voltage across the FEL and SEL is equivalent to the stacked voltage from all stages of the multi‐stage capacitive voltage booster. Thus, the developed TENG device controls the voltage output through a series capacitive voltage control mechanism and is thus named SCVC‐TENG. A model of the SCVC‐TENG with nodes and its equivalent circuit is shown in Figure S1a,b, respectively.

Thus, the SCVC‐TENG achieves power enhancement through the synergistic integration of floating‐electrode voltage multiplication, a capacitive charge reservoir mechanism, and a spark gap (Figure 1b). The SCVC‐TENG's measured output voltage, ∼5 kV (at 3 Hz), as exhibited in Figure 1b, is about 385% higher than the max voltage (V_max_) of the conventional TENG and 263% higher than its peak‐to‐peak voltage (V_pp_). As shown in Figure 1d, the peak output current across the load resistor (R = 1 MΩ) is ∼4.9 mA, around 175 times higher than the peak current of the conventional TENG. At a load resistance of 100 Ω, the SCVC‐TENG achieved a high pulse‐power density of ∼7.3 MW/m^2^ (instantaneous power of ∼18.25 kW), as shown in Figure 1g. This corresponds to a high pulse‐current of 13.4 A, which is achieved with an effective area of 0.0025 m^2^, as shown in Figure 1g. Such high instantaneous high power can be utilized for powering high‐power lasers [25]. This power density of SCVC‐TENG significantly surpasses that of previously reported contact‐separation mode TENG devices (Figure 1e) [1, 26, 32, 33, 34]. Furthermore, as shown in Figure 1f, although the charge output remains moderate, the current density achieved also exceeds that of existing contact‐separation mode TENG devices [1, 26, 32, 35]. In addition, this instantaneous high power output and current density of the SCVC‐TENG are comparable to the state‐of‐the‐art TENG devices, as shown in Figure S2 [1, 25, 26, 32, 36, 37, 38]. These results underscore the exceptional performance of the SCVC‐TENG and highlight the need for further optimization strategies aimed at enhancing charge output while maintaining high power and current densities. Furthermore, the instantaneous power density and current at high load resistances are shown in Figure S3a, and the observed voltage output across all resistances (100 to 100 MΩ) is shown in Figure S3b. As the resistance increased, both the power density and current decreased, whereas the voltage increased. However, even at 100 MΩ resistance, it showed a power density of 99.2 W/m^2^ and a current density of 19.9 mA/m^2^. In addition, the average power generation capability of the developed SCVC‐TENG is checked, and the resulting output is displayed in Figure S4. The average power density is measured by P_avg_ = $1/t\int_{0}^{t}\left(V{\left(t\right)}^{2}/R\right).dt$ utilizing one minute voltage obserevd across varying loads as shown in Figure S4b,c. The highest P_avg_ observed was 760 mW/m^2^ at a load of 100 Ω, and the lowest was 485.6 mW/m^2^. A comparison of the average power density and the energy efficiency of the SCVC‐TENG with previously reported contact‐separation mode TENG literature is shown in Table S1. Further, in an exhibition of the high‐pulse power output, our SCVC‐TENG, when coupled with EM, powered a combined rated capacity of 221 W LED bulbs set as shown in Video S2. This confirms the developed SCVC‐TENG's capability to power high‐power electronics in pulse mode. In addition, SCVC‐TENG, when combined with an EM system, can continuously drive an LED and a hygrothermograph.

## 3 The Working Mechanism of the Capacitive Charge Reservoir‐Enabled SCVC‐TENG Depending on the Gap of Spark Switch

The working mechanism of SCVC‐TENG varies with the gap of the spark switch. It is conjectured that the basic working mechanism of SCVC‐TENG comprises 6 stages, as illustrated in Figure 2a,b. Depending on the spark gap distance, the SCVC‐TENG can be utilized for a frequent‐discharge, charge‐pumping mode or an infrequent, high‐amplitude pulse mode. Frequent‐discharge, charge‐pumping mode can be achieved with a low spark gap, while a large spark gap focuses on high‐amplitude pulse mode output. In both cases, all layers are separated in the initial State (before the contact). In this state, all layers are fully charged from the previous cycle and at electrostatic equilibrium (Step VI). In step I, the FEL touches the top FTL, i.e., the TPU/G composite, starting a capacitive chain (Figure 2a, step I). In step II, the FEL and TPU/G assembly contacts the bottom FTL, i.e., the PTFE sheet. In this stage, true charge transfer occurs. PTFE sheet gains negative surface charges. and TPU/G sheet gains more positive surface charges (Figure 2a, step II). These charges are now bound, not free to move, creating an internal electric field. In this state, the whole system is in a non‐equilibrium state. The negative charges on FEL, rather than immediately discharging through an external circuit, these induced charges accumulate at the CCRs, specifically, on the electrode surfaces separated by small air gaps. In stage III, the entire top assembly (FEL + TPU/G + PTFE) now contacts the bottom electrode layer (SEL). This sets up a full multi‐layer capacitive system. However, the developed field is not enough to cause an air breakdown across the spark switch. Thus, at this stage, as the vertical gap between the FEL and SEL becomes lower than the sum of the spark gap and the CCR air gaps, it leads to a spark discharge between the FEL and SEL to neutralize the whole system, as illustrated in Figure 2a, step III. At this point, there's maximum field coupling across all layers; however, it is very clear that there is still no adequate charge flow across the external terminals, as observed voltages in two cases of low spark gap and large spark gap during this first stage of sequential contact, as exhibited in Figures 2c,d, stage 1. In further stage 2, the assembly starts to separate in reverse order, i.e., in step IV, the entire top assembly (FEL + TPU/G + PTFE) lifts off from the SEL. The capacitance between the SEL and PTFE layers decreases rapidly. When the spark gap is small (low spark gap), at this step, as the tribo‐charges are still present, and the capacitive coupling is broken, the voltage between electrodes is raised sharply since the capacitance decreases rapidly (following Equation 2). The resulting electric potential difference drives electrons to flow through the external circuit to restore electrostatic equilibrium as the spark gap is low. This generated electrical potential, V_TENG_, is higher than the breakdown voltage V_AB_ (between plate A and plate B of the spark gap switch), causing two electrostatic breakdowns across the spark gap as illustrated in Figure 2b(i), step IV and 2c stage 2. On further steps V and VI, also, the separation of each layer causes the capacitance of that particular interface to reduce rapidly, leading to electrostatic breakdowns across the spark gap (Figure 2b(i), steps V and VI). However, these two steps are only capable of generating a single spark at a time (Figure 2c, stage 2), thus producing a total of 4 voltage peaks in a single contact separation. Hence, the low‐spark gap focuses on efficient charge transfer with frequent‐discharge rather than high‐power output and thus can be utilized for continuous charging mode with the integration of energy management, and in turn charging capacitors and powering low‐power electronics (as shown in Figures 5 and 6).

Whereas when the spark gap is large, in steps IV and V (Figure 2b(ii)), as the capacitances between triboelectric layers decrease rapidly as air gaps increase, which causes voltage to rise sharply across the entire series‐coupled system. The CCR air gaps function as capacitive energy reservoirs with capacitance C_CCR_ = ε_0_A/d_gap_. These capacitances store charge incrementally as the separation progresses, building up voltage across the CCRs without triggering discharge. In this case, the spark gap does not see the instantaneous TENG voltage, but it sees the accumulated voltage across the charge reservoirs (following Equation 3). Critically, the charge stored in the CCRs cannot immediately escape, and it must overcome the breakdown voltage of the spark gap. Thus, only minor induced charge flow is observed in steps IV and V, as evident from Figure 2d, stage 2. No spark is observed at these stages since the developed field is not enough to cause an air breakdown across the spark gap. This threshold‐gating mechanism ensures that energy release occurs only when accumulated voltage is sufficient for high‐power delivery.

Thus, in the final step (step VI), V_accumulated_ across the CCRs exceeds the breakdown threshold V_BD_ of the spark gap, rapid discharge occurs, delivering the stored energy to the load as displayed in Figure 2d, stage 2. The discharge time (nanoseconds to microseconds, depending on load) is much shorter than the charge accumulation time (milliseconds for the full contact‐separation cycle), resulting in high instantaneous power despite moderate charge accumulation. Also, a large spark gap is only capable of generating a single high‐amplitude, abrupt voltage peak in a single contact‐separation cycle. Thus, this can be utilized for pulse‐mode high‐power generation (as shown in Figures 4f and 6).

A theoretical model for the SCVC‐TENG to give the V‐Q‐x relationship (V = Voltage, Q = Charge, x = total air gap) is derived by modelling the system as a stack of capacitors (Figure S1a,b) with fixed surface charges (σ) due to the triboelectric effect, and is given in detail in Note S1. The obtained V‐Q‐x relationship is:

(4) $$V\left(t\right)=\frac{-Q\left(t\right)}{C_{eff}\left(t\right)}+\frac{\sigma A\;}{C_{eff}\left(t\right)}$$

The obtained *V_oc_* across the triboelectric capacitor stack (Node 1 to Node 6 from Figure S1b) from the V‐Q‐x relationship is:

(5) $$V_{oc}=\sigma \left[\frac{d_{air}\left(t\right)}{\varepsilon_{0}}\right]$$

However, since the charge discharge in this investigation is caused by an air breakdown across the spark gap (Figure 2), the voltage, current, and power output follow a field‐dependent discharge mechanism. Based on this mechanism, air breakdown occurs when the electric field across the TENG electrodes, i.e., *E_TENG_* exceeds the dielectric breakdown strength (*E_BD_*) of the medium between the spark gap, i.e., air in this study (*E_BD_* ∼3 MV/m). In addition, since CCR 1 and CCR 2 are designed with capacitance C_CCR_ ≫ C_eff_ (t) across the operating range, the voltage division across the CCR stage is negligible, and the full V_oc_ is effectively available across the spark gap. Thus, according to these conditions and Equation S4b in Note S1:

(6) $$E_{TENG}=\frac{V_{oc}}{d_{S1+S2}}=\frac{V\left(t\right)}{dt}\;\geq E_{BD}$$

whereas *d*_*S*1 + *S*2_ is the sum of the small air gaps of CCR 1 and CCR 2 (Figure S1a), and *dt* is the air gap of the spark switch. Thus, the modified voltage equation just before the spark discharge, by considering Equation (6), can be given by:

(7) $$V\;\left(t\right)=E_{BD}\;\cdot \;dt$$

Further, from Equation 6, we have:

(8) $$V\;\left(t\right)=\frac{V_{oc}}{d_{S1+S2}}\;\cdot \;dt$$

hence, the voltage output by the SCVC‐TENG is directly proportional to the air gap of the spark switch gap and *V_oc_*. Thus, it is in turn directly proportional to *d_air_* (*t*) (Equation 5). As *d_air_(t)* increases during the separation stage, *C_eff_(t)* decreases rapidly. This causes a sharp rise in *P(t)*, since *P* (*t*) =  *V*(*t*) ·  *I*(*t*). Thus, the high pulse‐power density of ∼7.3 MW/m^2^ (Instantaneous power ∼ 18.25 kW), as shown in Figure 1g, corresponds to a current density of ∼5.36 kA/m^2^. This is ∼ 5.93 × 10^5^ and ∼ 2.55 × 10^5^ times that of the highest power density (12.3 W/m^2^) and the current density (0.021 A/m^2^), respectively, generated by the CCS‐TENG design (Figure S5). In addition, from 100 Ω to 10 kΩ of resistance, our SCVC‐TENG showed an excellent average power density of 2.86 MW/m^2^.

## 4 Performance of the SCVC‐TENG

It is conjectured that the outstanding performance of the SCVC‐TENG can also be attributed to the contrasting electron affinities of the triboelectric layers, namely, the TPU/G composite and PTFE sheets. The incorporation of graphene into the TPU matrix enhances its triboelectric behavior by the formation of micro‐capacitor structures within the composite due to graphene's high aspect ratio and conductivity [39]. Initially, a CCS‐TENG design was employed to evaluate TPU/G composites with varying graphene concentrations systematically to determine the optimal graphene loading for triboelectric performance. The working mechanism of the CCS‐TENG design is illustrated in Figure S6, and the empirical waveforms of voltage, current, and charge output are exhibited in Figure S7. Among them, the TPU/G composite containing 2 wt.% graphene (TPUG2) exhibited the highest output, delivering a peak‐to‐peak open‐circuit voltage (V_oc_) of 2.39 kV at 9 Hz, a short‐circuit current of 84 µA, and a transferred charge of 70 nC. The comparative performance results are presented in Figure S8 and discussed in detail in Note S2. Further, the corresponding contact potential difference (CPD) values for TPU/G2 and Teflon (commonly used as an indicator for determining the differences between the tribo‐pairs) were determined using the KPFM studies. TPU/G2 registered a max CPD value of 303 mV, while the CPD value for Teflon was −9.99 V. This indicated that both the tribo‐layers selected for the current system are electronically very different and warrant the contact electrification process to a greater extent. The corresponding KPFM images are shown in Figure S9. Consequently, the TPUG2 composite was selected for fabricating the SCVC‐TENG.

**FIGURE 3:**
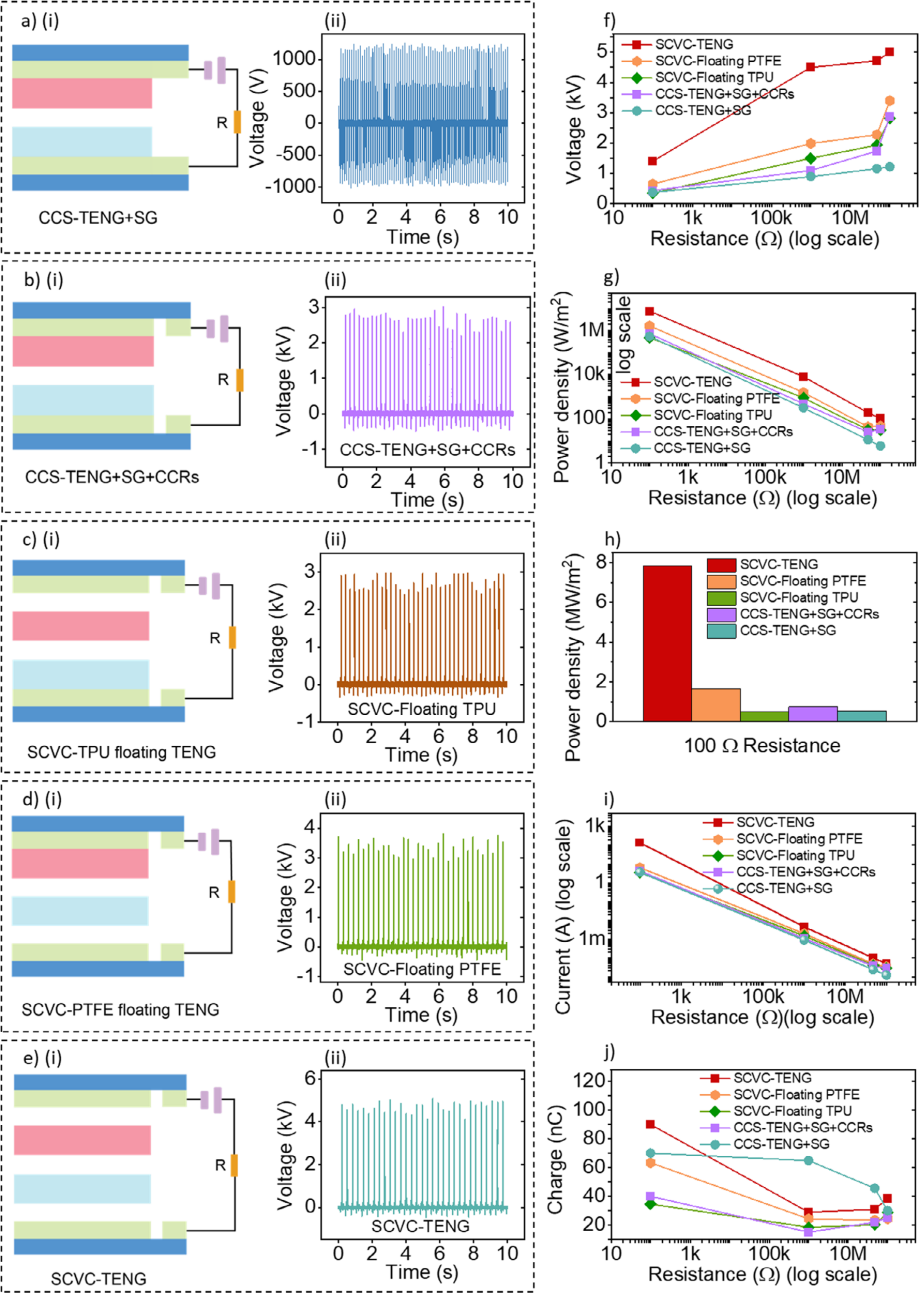
The design and its corresponding voltage output when monitored for 10 s: (a) CCS‐TENG+SG, (b) CCS‐TENG+SG +CCRs, (c) SCVC‐TPU floating TENG, (d) SCVC‐PTFE floating TENG, (e) SCVC‐TENG. Quantitative comparison of the key performance indicators at different load resistances of 100 Ω, 1 MΩ, 47.4 MΩ, and 100 MΩ: (f) Voltage, (g) power density, (h) power density at 100 Ω, (i) Current, and (j) Charge.

To systematically evaluate the advantages of the SCVC‐TENG architecture, several alternative structural designs were fabricated and tested, as illustrated in Figure 3a–e, namely, CCS‐TENG+SG (CCS‐TENG with spark gap), CCS‐TENG+SG+CCRs, SCVC‐TPU floating TENG, and SCVC‐PTFE floating TENG. These control configurations were developed by varying the electrode connectivity, floating layer arrangement, and spark‐gap coupling, allowing a direct comparison of design‐dependent output performance. The corresponding open‐circuit voltage outputs for each design are displayed in Figures 3a(ii)–e(ii). This displayed observation highlights the clear superiority of the SCVC‐TENG, which delivers substantially higher voltage output (∼5 kV) compared to all the control devices (∼1.2 to 3.5 kV). This performance enhancement can be attributed to the sequential contact and capacitive voltage coupling mechanism, which amplifies voltage accumulation across the floating dielectric assembly before discharge, and the introduction of the CCRs, which act as charge reservoirs before the spark gap switch, which prevents direct influence of the spark gap on TENG. To further substantiate this claim, a quantitative comparison of the key performance indicators, such as voltage, power density, current, and charge, is examined at different load resistances of 100 Ω, 1 MΩ, 47.4 MΩ, and 100 MΩ, capturing both low‐ and high‐impedance scenarios. The corresponding empirical waveforms of individual peaks are displayed in Figures S10–S14. The comparative voltage, power density, current, and charge output from these observations are exhibited in Figures 3f–j. Clearly, at all resistances, the SCVC‐TENG produced high voltage in comparison to all the control configurations (Figure 3f). Similarly, the SCVC‐TENG also exhibited higher power density at all resistances (Figure 3g). Figure 3h shows that the SCVC‐TENG generated a power of 7.3 MW/m^2^ at 100 Ω load, whereas all the other architecture designs generated < 2 MW/m^2^ at the same resistance. This observation strengthens the claim of the clear superiority of the SCVC‐TENG. The observed current also followed a similar trend, with SCVC‐TENG leading at all load resistances (Figure 3i).

However, the charge output showed a different trend where SCVC‐TENG exhibited the highest at 100 Ω and 100 MΩ, whereas at 1 MΩ and 47.4 MΩ, ‘SCVC‐TENG+SG’ exhibited the highest. This increase in charge for the ‘CCS‐TENG+SG’ can be attributed to the formation of a spark during both stages, i.e., contacting and separation (Figure 3a(ii)), unlike the DC voltage observed in SCVC‐TENG, i.e., only during separation (Figure 3e(ii)). Thus, the charge loss into the air due to the waiting period of spark formation will be lower in ‘CCS‐TENG+SG’. To further the comparison, an SCVC‐TENG with dimensions of 10 × 8 cm was fabricated (termed as ‘Large SCVC‐TENG’ from here on) and analysed for its power density, current, and charge output across varying loads. The observed output is displayed in Figure S15. Clearly, with an increase in the area of the triboelectric layers, the power density, current, and charge output increased significantly, confirming the advantages of the CCR and floating design structure (see Note S3 for further details). Further, since the device operates in high‐field, the volumetric dielectric heat generation under the device's operating field is estimated and discussed in detail in Note S3. It confirms a substantial safety margin below the material's breakdown strength and negligible areal heat generation rate of 27 mW/m^2^ of the elastomeric layers (Note S3).

In addition, a theoretical analysis with the Field‐Dependent Discharge (FDD) mechanism for peak current and peak power at varying loads of 100 Ω, 1 MΩ, 47.4 MΩ, and 100 MΩ is carried out and is discussed in Note S4, following Equation (7) derived utilizing the equivalent circuit displayed in Figure S1b. The comparison of the theoretical and experimental peak current and peak power output is given in Table S2. It is also plotted and displayed in Figure S17. Even though the current is not varying as much, it is very clear from this comparison that at low external load resistances, the experimental power deviates very much from the theoretical value, as compared to the high external load resistances. It is a virtue of the voltage capping at low loads (*R_load_* = 100 Ω). However, at higher loads (*R*_*load* _< 1 MΩ) SCVC‐TENG showed higher charge transfer efficiency (η  > 50%). It exhibited 74% efficiency at a high load of 100 MΩ (See Notes S4 for more details) demonstrating that the equivalent circuit is not merely descriptive but predictive, and identifying high‐resistance operation as the regime where the model's practical design utility is strongest.

All of the above findings reveal that the integration of floating electrodes and triboelectric layers, the sequential capacitive coupling structure, and the introduction of CCRs significantly enhance power output and improve charge to an extent as compared to the conventional design TENG. This systematic analysis confirms that the SCVC‐TENG effectively bridges the performance gap between conventional CS TENGs and high‐power pulse‐generation systems.

Thus, further, the performance of SCVC‐TENG was carried out at varying spark gaps and working frequencies.

**FIGURE 4:**
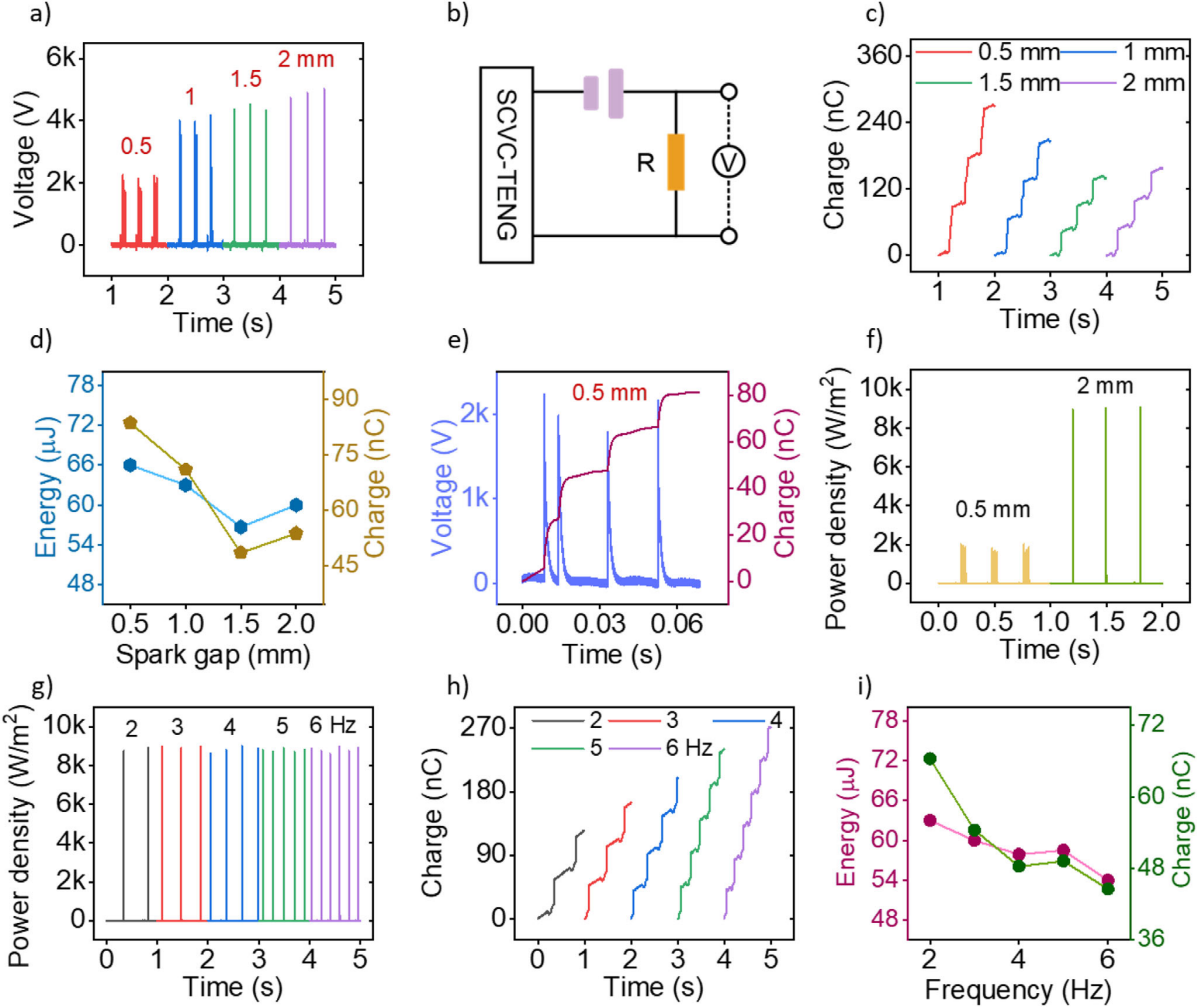
(a) Turn‐on voltages obtained by varying air gaps of the spark switch from 0.5 to 2.0 mm. (b) Circuit illustration of the charge measurement setup. (c) Observed charge transfer by varying the spark gap from 0.5 to 2.0 mm. (d) Charge and energy accumulated per cycle at corresponding spark gaps. (e) Multiple discharge peaks and the corresponding charge discharge observed at a 0.5 mm spark gap. (f) Instantaneous power density observed at 0.5 and 2 mm spark gap. (g) Instantaneous power density observed with varying frequency. (h) Charge output observed with varying frequency. (i) Charge and energy per cycle with varying frequency.

Figure 4a displays the different turn‐on voltages (also termed as V_oc_) in the range from 2 to 5 kV obtained by systematically varying the air gaps of the spark switch from 0.5 to 2.0 mm. A detailed image and working mechanism of the spark switch utilized in this study are illustrated in Figure S18a and Note S5. Note that a parallel plate design is used for the spark switch, owing to its lowest leak current and highest stability compared with other switch designs, such as plate to tip, tip to plate, and tip to tip [1]. Figure S18b shows the observed spark of electric breakdown between the parallel plates. As the displacement increases from 0.5 to 2 mm, the output voltage exhibits a nonlinear but monotonic increase in amplitude. This behavior is consistent with fundamental breakdown dynamics of air, which obey Paschen's Law, which states breakdown voltage, *V_break_* [40],

(9) $$\;V_{break}=\frac{aPd}{\ln\left(bPd\right)-\ln\left[\ln\left(1+\frac{1}{\gamma }\right)\right]}$$

where *a, b*, and γ are constants. *P* is the atmospheric air pressure, and *d* is the air gap between the spark switch plates A and B. Thus, *V_break_* is directly proportional to the spark switch gap. The spark gap introduces a threshold condition where charge accumulation on the TENG continues until the local electric field across the gap surpasses the breakdown strength of air (∼30 kV/cm under standard atmospheric conditions). At smaller spark gaps, discharge events occur more frequently, leading to shorter charging times and lower peak voltages. The result is repetitive, lower‐amplitude multiple voltage (∼2 kV at 0.5 mm) spikes as energy is discharged before reaching high field intensity. At higher spark gaps, the breakdown threshold increases (∼5 kV at 2 mm). The system accumulates more energy before discharge, producing sharper, higher‐amplitude voltage peaks. However, the time required to reach this threshold grows, leading to less frequent but high‐power pulse peaks. The charge transfer with the corresponding spark gap is recorded at a medium load resistance of 50 kΩ and displayed in Figure 4c. The electrical circuit followed for this measurement is illustrated in Figure 4b. The observed charge accumulation behavior reveals that the total transferred charge per cycle decreases as the spark gap increases initially, and after a particular gap, it starts to increase. This trend can be attributed to two interconnected physical effects: delayed breakdown at higher spark gaps and capacitive discharge efficiency. As the spark gap increases initially, the electric field required to trigger air ionization becomes larger [41]. Consequently, the TENG system must reach a higher potential before the breakdown condition is met. During this waiting phase, less energy is converted into charge motion, and energy is instead stored as an electric field across the spark gap. This results in less frequent and more abrupt discharge events, reducing the effective charge transfer through the external circuit due to combinations of delayed breakdown, parasitic capacitance, and leakage through air [41, 42, 43]. However, after a particular spark gap, since the SCVC‐TENG behaves as a high‐impedance capacitive source, its output charge transfer is mediated by capacitive build‐up until the breakdown field is achieved across the spark gap, which can be given as: [44]

(10) $$\;Q_{TENG}=C_{eff}\left(t\right)\;.\;V_{break}$$

Thus, with an increase in the *V_break_* on increasing the spark gap, the stored charge available at breakdown also increases. This supports the observation made in Figure 4d, which displays the corresponding energy transfer on varying the air gaps of the spark switch. It follows a similar trend to charge transfer but with less steepness. This can be understood from the general equation for energy, *E*: [45]

(11) E=∫V.dQ

hence, the loss in charge is partially compensated by an increase in voltage, which flattens the energy curve.

These results emphasize that spark gap tuning acts as a threshold filter that can guide the SCVC‐TENG to two different application scenarios: a) continuous energy harvesting at a lower spark gap for charging mode, which prioritizes cumulative charge transfer, and b) at larger spark gaps, a high instantaneous pulse high‐power mode, which prioritizes high‐voltage events over cumulative charge transfer. As shown in Figure 4e, the SCVC‐TENG at a 0.5 mm spark gap exhibits highly favorable characteristics for energy harvesting. Each contact‐separation cycle consistently initiates discharge events at relatively lower voltages (∼2 kV), allowing consistent triggering with each mechanical cycle. This maximizes spark discharge events, ensuring reliable charge flow (∼83 nC at 50 kΩ load). This stepwise charge delivery demonstrates the capability of the system to function as a repetitive charge pump, making it well‐suited for energy harvesters that prioritize stable and repetitive energy delivery over high instantaneous power. However, as shown in Figure 4f, increasing the spark gap in the SCVC‐TENG leads to a marked enhancement in instantaneous power density (shown power density is when a 1 MΩ load is provided). While smaller gaps facilitate frequent but lower‐power pulses, larger gaps accumulate more charge and discharge it rapidly once the higher breakdown voltage is reached. This results in short, high‐energy pulses that can even exceed 8.5 kW/m^2^ on a load of 1 MΩ at a 2 mm spark gap. Such performance is particularly advantageous in applications demanding high instantaneous pulse power. In addition, Figure 4g shows that the power density output of the SCVC‐TENG remains relatively invariant with increasing contact‐separation frequencies, demonstrating its ability to deliver consistent instantaneous pulse power for a range of working frequencies. However, Figure 4h,i show a higher reduction in charge transfer per cycle with increasing frequency and a marginal reduction in energy, likely due to reduced charge accumulation time. This trade‐off suggests that while cumulative energy harvesting performance diminishes slightly at higher working speeds, the system remains well‐suited for frequency‐independent high‐voltage pulsed applications in scenarios where the working frequency may fluctuate due to user or environmental factors. Further, the energy transformation efficiency of the SCVC‐TENG from mechanical power to electrical power is examined as shown in Note S6 and is found to be 70.1%. In addition, to examine the capability of SCVC‐TENG in a real‐world applicability, its performance with varying humidity of 45%, 60%, and 75% relative humidity is examined and displayed in Figure S19. As expected, with increase in the humidity, the voltage output decreased. Till 60% RH, the reduction was minimal (< 5%), but on increasing to 75% RH, the voltage reduced 20% as compared to voltage at 45% RH. However, this confirms the developed SCVC‐TENG is capable of operating in a range of humidity, even with reduced voltage output, confirming the real‐world applicability of the SCVC‐TENG.

## 5 Energy Harvesting Utilizing an Energy Management (EM) System for Continuous Charging Mode

**FIGURE 5:**
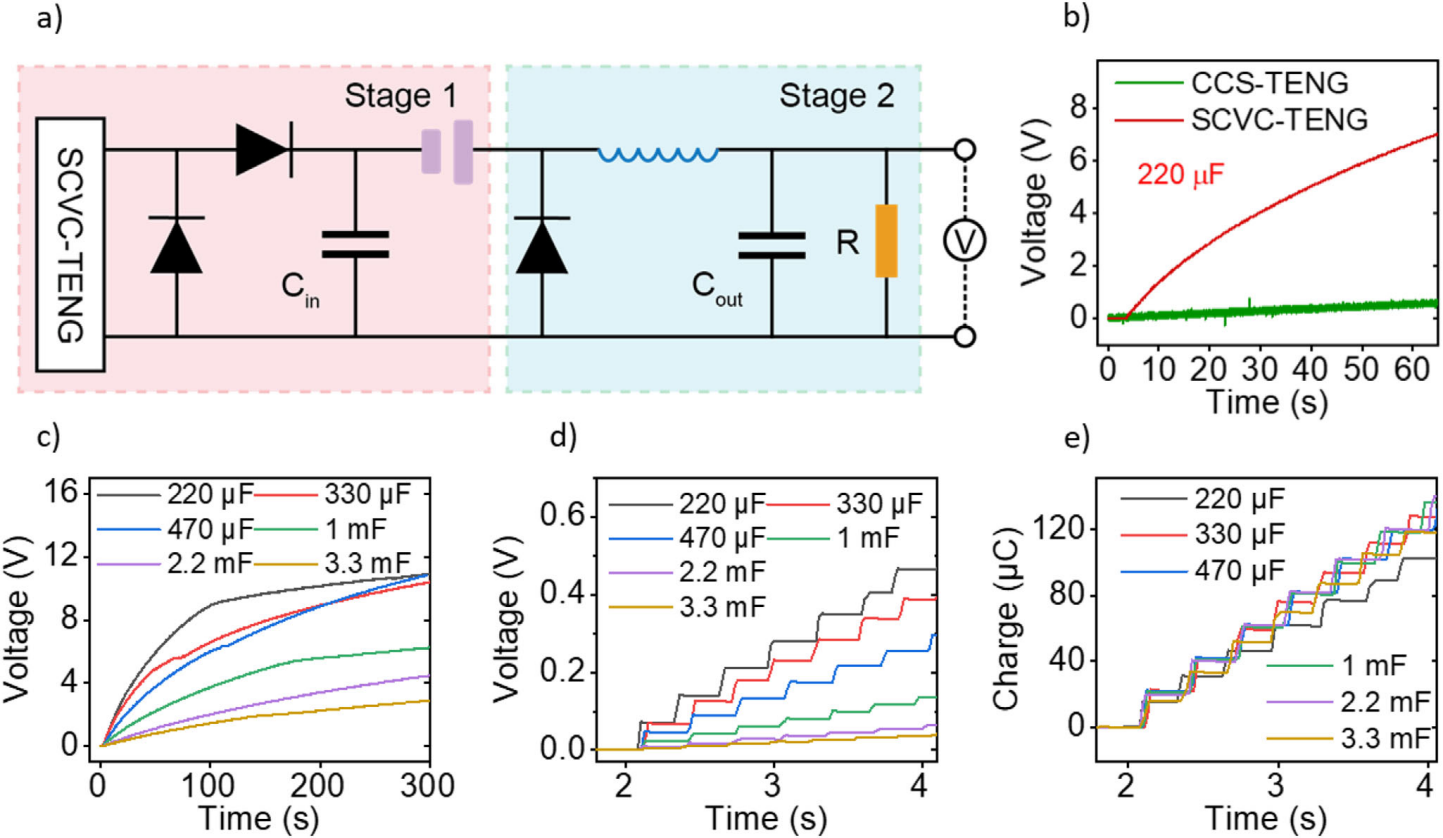
(a) Schematic illustration of energy management. (b) 220 µF Charging comparison of SCVC‐TENG with CCS‐TENG. (c) Charging curves of different capacitors. (d) Voltage accumulation per cycle in capacitors. (e) Charge accumulation per cycle in capacitors.

Following Zhang's work [32], an inductor‐based EM system is utilized in this study. The circuit diagram of the EM is illustrated in Figure 5a. It is divided into two stages. The first stage acts as a stable high‐voltage source, and the second stage consists of a matched inductor with a flyback diode to charge the capacitors. A half‐wave rectification is adopted here as a useful method to generate a stable high voltage. Also, a spark gap of 0.5 mm is maintained in this section of the study to maximize spark discharge events and ensure reliable charge flow, following the observation made in section 4. While introducing the EM system, the spark gap was placed systematically after the C_in_ to ensure a stable voltage and charge output. The voltage and charge output after stage 1 of the EM system are displayed in Figure S20. It demonstrated a voltage output of ∼6 kV (Figure S20c), and enhanced charges of ∼96 and ∼60 nC, at 0.5 and 2 mm spark gaps, respectively, at 50 kΩ loads (Figure S20b,c). This performance enhancement is attributed to C_in_ storing the extra charge of SCVC‐TENG when the voltage is less than V_break_. Further, the cyclic stability of the output performance of Stage 1 is displayed in Figure S20d, which confirms the stable output of Stage 1 of the EM system. In addition, the voltage output of the CCS‐TENG with first stage of EM is examined for comparison and is showed in Figure S21. It generated a maximum peak voltage of ∼2.45 kV, which is 59% less than the voltage output of SCVC‐TENG.

Since the low spark gap (0.5 mm) transfers the highest charge, it is used for continuous charging mode, and Figure 5b displays a 220 µF capacitor charging performance of the SCVC‐TENG and the CCS‐TENG over a 60 s duration at a 4 Hz working frequency. The SCVC‐TENG exhibited a significantly higher voltage accumulation compared to the CCS‐TENG. The capacitor voltage charged by the SCVC‐TENG rose rapidly and continued to increase steadily, reaching a value of ∼7 V, whereas the CCS‐TENG capacitor voltage was only ∼0.5 V, a 1400% difference in accumulated voltage. This result directly translates into superior charge transfer and storage capability of the SCVC‐TENG in combination with the EM system, underscoring its efficient charge accumulation behavior during repetitive mechanical stimuli. To further assess the energy harvesting capability of the SCVC‐TENG, various capacitors with capacitance values ranging from 220 µF to 3.3 mF were charged using the harvested output, and their voltage accumulation was monitored over 5 min (Figure 5c). The voltage output demonstrates the superior charging capability of the SCVC‐TENG across all tested capacitor values. For the capacitor (220 µF), the voltage rose rapidly to ∼9 V within 90 s, indicating efficient energy harvesting even at a low working frequency of 4 Hz. Within 5 min, the 330 µF and 470 µF capacitors reached voltages of approximately 10.2 and 10.8 V, respectively. For larger capacitances, such as 1, 2.2, and 3.3 mF, the voltage still increased significantly, reaching 6.35, 4.42, and 2.87 V, respectively. These results emphasize the capability of the SCVC‐TENG to effectively harvest energy from relatively low working frequencies. As compared to the high power density output, this charging efficiency is only comparable to the high‐performing TENGs. This is a virtue of the low output charge of the SCVC‐TENG because of the transient charge transfer at the spark gap switch. Thus, further studies should be done to enhance the charge output. Further, Figures 5d,e display the voltage and charge accumulation per contact‐separation cycle, respectively. As seen in the voltage profile, with each contact–separation cycle, the output voltage increases in a clear stepwise fashion for all capacitors tested. Notably, the 220 µF capacitor reaches the highest voltage (∼100 mV) per cycle, followed by 330 µF and 470 µF capacitors, while larger capacitors (2.2 and 3.3 mF) show comparatively slower voltage rises, as expected due to their greater energy storage capacity. Similarly, the cumulative charge transferred into the capacitors also increases per cycle. Capacitors 470 µF and 1 mF showed the highest charge with 20 µC per cycle (charge density ∼ 8mC/m^2^). Whereas, the remaining capacitors showed a lower charge accumulation per cycle. The results demonstrate that even at low working frequencies, the SCVC‐TENG exhibits strong energy harvesting characteristics through consistent voltage buildup and cumulative charge transfer, validating its suitability for intermittent mechanical motion environments such as human motion or low‐speed vibrational sources.

## 6 Applications of SCVC‐TENG

**FIGURE 6:**
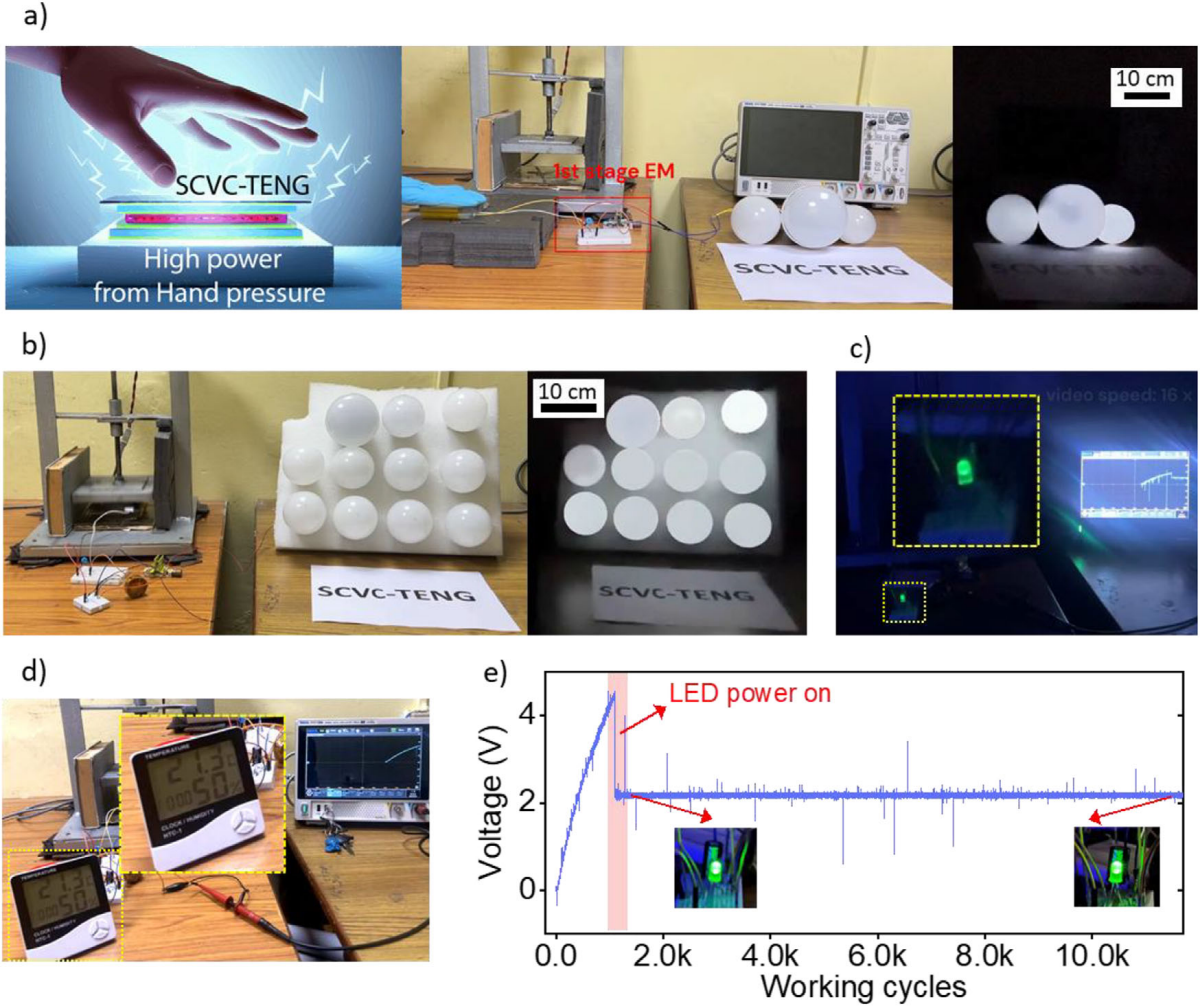
(a) Powering 70 W LED bulbs with low hand pressure. The inset shows the circuit followed for directly powering the bulbs. (b) 221 W combinely rated bulbs powered after the second stage of EM. (c) Powering a green LED continuously with EM.  (d) Driving a hygrothermograph continuously with EM. (e) Voltage output corresponding to continuously powering an LED for more than 11,500 cycles.

To validate the real‐world applicability of the SCVC‐TENG, we demonstrate its exceptionally high‐power performance by lighting commercial 70‐W combined rated LED bulbs with the very low‐press‐and‐release cycles of the TENG device by human hand with an effective area of 0.0025 m^2^ (Figure 6a and Video S1). As discussed earlier, these high‐power bulbs are lit at a spark gap of 2 mm. As shown in Figure 6a, a single press‐and‐release cycle of the device by hand (with an average force of 8 to 13 N at a 1 Hz frequency) is sufficient to generate the required power to illuminate multiple high‐wattage bulbs instantaneously. This demonstration highlights the capability of the SCVC‐TENG to deliver raw, high‐voltage, high‐current pulses with low press‐and‐release cycles by human hand, suitable for directly driving power‐intensive electronic loads. In addition, leveraging the SCVC‐TENG with full EM, we successfully lit up 11 commercial LED bulbs, combinely rated an enormous 221 W, with an effective area of 0.008 m^2^ (Figure 6b and Video S2). The power transfer efficiency during this lighting of combined 221 W rated LED bulbs was found to be 59.7% (Note S7). The same setup of 221 W LED bulb's combination was also lit up with only the first stage of EM (Video S3). Evidently, with the full EM, the bulbs lit up with higher intensity. The effective lighting of a 221 W LED bulb combination is significantly higher than the previous reports. Further, the continuous powering capability of SCVC‐TENG with EM is demonstrated by powering a µW‐power green LED continually at 4 Hz with an effective area of 0.0025 m^2^ with a lower spark gap of 0.5 mm (Figure 6c and Video S4). In addition, a commercial hygrothermograph is also continually driven at 4 Hz with the same effective area (Figure 6d and Video S5). The corresponding charging curves of a 2.2 mF capacitor for continuously driving the LED and hygrothermograph are displayed in Figures S23a,b. Finally, the long‐term working stability of the SCVC‐TENG in combination with the EM is carried out by continually driving the µW‐power green LED for ∼50 min, and the resulting voltage is displayed in Figure 6e. The resulting voltage curve shows a 99.3% retention in voltage even after 11 500 working cycles. The inset of Figure 6e shows no noticeable change in the intensity of the LED even after 11 500 working cycles as compared to the beginning of powering.

## 7 Conclusions

In summary, we have developed and demonstrated a high‐performance Series Capacitive Voltage Control Architecture Triboelectric Nanogenerator (SCVC‐TENG) that addresses the longstanding limitations of conventional contact‐separation mode TENGs. By integrating a strategically engineered floating triboelectric assembly composed of a graphene‐enhanced TPU (TPU/G) and PTFE sheet, along with an automatic spark‐gap, and a capacitive charge reservoir, the SCVC‐TENG achieves a substantial enhancement in both current and power output. The device delivers a current density of ∼5.36 kA/m^2^ and a peak pulse power density of ∼7.3 MW/m^2^. This enables it to power high‐load devices such as 70 W LED bulbs with low‐pressure hand‐press and release, and a high power of 221 W LED bulbs using EM. Furthermore, the introduction of the inductor‐based EM circuit allows the SCVC‐TENG to operate in a controlled, continuous mode suitable for powering low‐power electronics such as an LED, and commercial hygrothermographs. Notably, long‐term cyclic tests demonstrated 99.3% output retention even after 11,500 cycles, establishing both electrical and mechanical stability of the system. Thus, this work introduces a transformative approach to enhance the practical utility of contact‐separation mode TENGs through floating‐layer capacitive amplification and intelligent structural design by introducing capacitive charge reservoirs for next‐generation self‐powered systems that address both high‐power pulse applications and low‐energy continuous needs. Our work thus redefines the capabilities of contact‐separation TENGs and sets a new benchmark for their integration into sustainable IoT and wearable electronic platforms.

## 8 Methods

### 8.1 Fabrication of SCVC‐TENG

The SCVC‐TENG consists of one floating electrode layer (FEL), one static electrode layer (SEL), and two floating triboelectric layers (FTL‐A and FTL‐B). The SEL, a 100 µm‐thick copper (Cu) tape of 5.1 × 5 cm, is adhered to the middle of a polyethylene terephthalate (PET‐1) sheet of 10 × 7 cm and another Cu tape (1 × 1 cm) with wires soldered to it is adhered at a gap of 0.2 mm from the SEL. The FTL‐A, a 400 µm‐thick PTFE film of size 5 × 5 × 0.04 cm, is adhered to the 0.5 cm length side of one end of PET‐1 and 0.5 cm of the other end to form an oval band‐like structure (see Figure S24a). Then, another PET sheet (PET‐2) of 14 × 7 cm is co‐axially and co‐centrally adhered to the bottom side of PET‐1. Now, FTL‐B, a thermoplastic polyurethane/graphene (TPU/G) composite of a 300 µm‐thickness and size of of size 5 × 5 cm, is adhered to PET‐2, in a similar manner FTL‐A was adhered to PET‐1, structurally to form a dual oval band‐like structure. Finally, FEL, another 100 µm‐thick Cu tape of 5 × 5 cm, is attached to the middle of PET‐3, another PET sheet of size 16 × 7 cm, and another Cu tape (1 × 1 cm) with wires soldered to it is adhered at a gap of 0.2 mm from the FEL. Then this combination was adhered to the already‐made double oval band‐like structure, in a way that the Cu electrode is inside the oval shape and faces the FTL‐B, as shown in Figure S24a, to form a triple oval band‐like structure. The sizes of all the control architectures, namely, CCS‐TENG, SCVC‐TPU floating TENG, SCVC‐ PTFE floating TENG are exhibited in Figures S24b‐d.

### 8.2 Characterizations

The fabricated TENG in contact‐separation motion is driven using an in‐house‐built tapper device. The voltage and current output are measured using a digital storage oscilloscope, Rigol DHO 1204, equipped with a 100 MΩ probe. The current and charge output of the CCS‐TENG (without spark gap and CCRs) is carried out using Keithley Electrometer 6517b. For all the other currents, including high‐frequency giant current measurement, voltage is probed across a known resistance, R, and the equation I = V/R is utilized. The charge Q output from the TENG is calculated from Q = ∫ Idt. The energy output is calculated by measuring the area of the voltage vs. charge (V‐Q) plot. For all TENG performance testing, the fabricated TENG device is affixed to a PMMA sheet, and a sponge, 15 mm thick, is adhered on top to ensure maximum contact between the triboelectric layers and electrode layers. Dielectric spectroscopy of the TPU/G composites and the PTFE sheet is carried out utilizing Novotherm Alpha. The Kelvin Probe Force Microscopy (KPFM) was done utilizing MFP‐3D Asylum Research, an Oxford Instruments Company AFM system. The COMSOL Multiphysics software is utilized to carry out the simulation investigations. The high‐voltage diode used in the half rectification is 2CL75 with an 18 kV breakdown voltage. For the flyback diodes, we used a R4000 capacitor. Moreover, our experiment was carried out in an ambient environment with humidity in the range of 50%–70% and temperature in the range of 25°C–30°C.

## Author Contributions

**Kinsuk Naskar**: writing – review and editing, resources, investigation, supervision. **Titash Mondal**: conceptualization, investigation, writing – review and editing, methodology, supervision, data curation, funding acquisition, project administration. **Ajay Haridas Cp**: conceptualization, methodology, validation, writing – original draft, formal analysis, data curation, investigation. **Prosenjit Das**: software, validation, visualization, supervision.

## Conflicts of Interest

The authors declare no conflicts of interest.

## Supporting information




**Supporting File 1**: advs77535‐sup‐0001‐SuppMat.docx.

**Supporting File 2**: advs77535‐sup‐0002‐VideoS1.mp4.

**Supporting File 3**: advs77535‐sup‐0003‐VideoS2.mp4.

**Supporting File 4**: advs77535‐sup‐0004‐VideoS3.mp4.

**Supporting File 5**: advs77535‐sup‐0005‐VideoS4.mp4.

**Supporting File 6**: advs77535‐sup‐0006‐S5.mp4.

## Data Availability

The data for all the experimental work is available from the corresponding authors based on reasonable requests.
