# Supplementary material for: Achieving High‐Instantaneous Pulse Power in Contact‐Separation TENG via Series Capacitive Voltage Control Architecture
Source: Adv Sci (Weinh). 2026 Sep 10:e77535. Online ahead of print. doi: 10.1002/advs.77535 (PMC13562781; doi:10.1002/advs.77535)
Supplement: Supplementary file 1 [file ADVS-9999-e77535-s006.docx]

**Supplementary Information**

**Achieving High-Instantaneous Pulse Power in Contact-Separation TENG via** **Series Capacitive Voltage Control Architecture**

*Ajay Haridas CP,^a^ Prosenjit Das,^b^ Kinsuk Naskar,^a*^ and Titash Mondal^a*^*

a Rubber Technology Centre, Indian Institute of Technology, Kharagpur, India- 721302

b Department of Electrical Engineering, Indian Institute of Technology, Kharagpur, India-

721302

E-mail: titash@rtc.iitkgp.ac.in ; knaskar@rtc.iitkgp.ac.in

**Figure S1.** a) Model of SCVC-TENG with nodes. b) Equivalent circuit of SCVC-TENG.

**Figure S2.** a) Comparison of the instantaneous power density and the matched impedance obtained in this work with other reports (the background gradient serves as an indicator of performance, with optimal values concentrated in the upper left region of the plot).^[1–6]^ b) Comparison of the instantaneous current density and the charge density obtained in this work with other reports (the background gradient serves as an indicator of performance, with optimal values concentrated in the upper right region of the plot).^[1–4,6,7]^

**Figure S3.** a) Power density and current at high load resistances (100 kΩ TO 100 MΩ). b) The voltage output observed across the complete external load resistances (100 Ω to 100 MΩ)

**Figure S4.** a) The average power density output of SCVC-TENG with varying loads. Representative voltage output of SCVC-TENG at: a) 100 Ω. b) 1MΩ.

**Table S1.** Comparison of average power density and energy efficiency of contact-separation mode TENG devices with SCVC-TENG

| Sl.No | Triboelectric materials | Average power density | Energy efficiency | Operating frequency | Reference |
| --- | --- | --- | --- | --- | --- |
| 1 | PTFE and copper | 291 mW/ m^2^ | Not reported | 3 Hz | ^[1]^ |
| 2 | PTFE and aluminium | 246 mW/ m^2^ | Not reported | 3 Hz | ^[4]^ |
| 3 | PTFE, PDMS, and aluminium | 532 mW/ m^2^ | Not reported | 4 Hz | ^[8]^ |
| 4 | Nylon and PTFE | 0.92 W/m^3^ | Not reported | 18 Hz | ^[9]^ |
| 5 | PET and aluminium | 768.5 mW/ m^2^ | Not reported | 40 Hz | ^[10]^ |
| 6 | PDMS, aluminium, and gold | Not reported | 22.6 % | 3 Hz | ^[11]^ |
| 7 | PTFE and aluminium | 89.4 mW/m^2^ | Not reported | 3 Hz | ^[12]^ |
| 8 | TPU/G and PTFE | 760 mW/m^2^ | 70.1% | 4 Hz | *This work |

**Note S1: Theoretical model of the SCVC-TENG**

We model the system as a stack of capacitors with fixed surface charges (*σ*) due to the triboelectric effect. **Figure S1A** shows the structure of SCVC-TENG. Two dielectric layers, a) TPU/G and b) PTFE sheets of thickness d_1_ and d_2,_ are floating in between two Cu electrode layers as shown in Figure 3a. The total air gap thickness between the top and bottom electrode layers is defined as ‘*d_air_(t)*’ (which is the sum of *x+y+z*). After the contact of all layers of SCVC-TENG as shown in **Figure 2a**, III^rd^ stage, both the top and the bottom triboelectric layers will have static triboelectric charges due to contact electrification. For simplicity, we assume the triboelectric charge density on the TPU/G layer as *+σ* (tribo-positive layer) and on the PTFE as *–σ* (tribo-negative layer). At the VI^th^ stage (**Figure 2b**), the two metal electrode layers will get polarized to mirror the static tribo-charge from the respective dielectric layer because of charge conservation.

To gain a comprehensive understanding of the electrostatic behavior and operational principles of the system, a node-based analytical approach is adopted.^[13–15]^ Given the excellent insulating properties of the dielectric layers and the characteristics of contact electrification, it can be inferred that the charges are uniformly dispersed across each surface at a macroscopic scale with minimal degradation.^[15]^ Consequently, the electric potential on each of the key conductive and dielectric interfaces, namely, top electrode, bottom electrode, and both the surfaces of top and bottom dielectric layers can be regarded as uniform. These six equipotential surfaces are thus treated as discrete electrostatic nodes within the analytical model, as shown in **Figure S1a**, where a node is a surface/volume with a certain electrical potential.^[13]^ The electrical line connection between every two nodes forms an equivalent capacitance between them. The corresponding equivalent circuit of the SCVC-TENG is shown in **Figure S1b**. The physical symbols utilized in this model are given below:

**Physical Symbols:**

Q_tribo_: Net triboelectric surface charge generated (fixed)

*C_eff_(t)*: Time-varying effective capacitance between electrodes

*V(t)*: Output voltage across terminals

*I(t)*: Current through external load

*ε₀*: Vacuum permittivity

*A*: Effective contact area

*d₁, d₂*: Dielectric thicknesses of TPU/G and PTFE dielectrics

*d_air_(t)*: Time-varying air gap during separation (sum of x+y+z)

*d_0_*: Effective dielectric thickness

$E_{TENG}$: Total electrical field generated by the TENG

d(t): Distance between the electrodes with time

*ε_r₁_, ε_r₂_*: Relative permittivity of TPU/G and PTFE dielectrics

*x, y, z*: the maximum air gap between the respective layers as shown in **Figure S1a.**

Now, the total capacitance between the two electrodes (Node 1 & 6) is the series connection of five capacitors from *C_1_* to *C_5_* (*C_eff_(t)*), which can be given by the following equation:

$\frac{1}{C_{eff}(t)}= \frac{1}{C_{1}}+\frac{1}{C_{2}}+\frac{1}{C_{3}}+\frac{1}{C_{4}}+\frac{1}{C_{5}}$	(1a)

Where:

$C_{2} = \frac{d_{1}}{\varepsilon_{0}\varepsilon_{r1}A}$	(1b)

$C_{4} = \frac{d_{2}}{\varepsilon_{0}\varepsilon_{r2}A}$	(1c)

And since the capacitors *C_1_, C_2_*, and *C_3_* are contributions of the respective air gaps, the sum *C_air_(t)*, of *C_1_, C_3_*, and *C_5_* can be given as:

$C_{air}\left( t \right)= \frac{d_{air}(t)}{\varepsilon_{0}A}$	(1d)

Thus,

$C_{eff}\left( t \right)=\left[ \frac{d_{1}}{\varepsilon_{0}\varepsilon_{r1}A} \right.+\frac{d_{2}}{\varepsilon_{0}\varepsilon_{r2}A}+\left. \frac{d_{air}(t)}{\varepsilon_{0}A} \right]^{-1}$	(2a)

Which can also be simplified to:

$C_{eff}\left( t \right)=\frac{\varepsilon_{0}A}{d_{0}+d_{air}(t)}$	(2b)

Where *d_0_* is the effective dielectric thickness, which is defined as the summation of all the thicknesses of the dielectric *d_i_* inside the air gap divided by its relative permittivity ε_ri_, as shown below:

$d_{0}= \sum_{i=1}^{n} \frac{d_{i}}{\varepsilon_{ri}}$	(2c)

And $d_{air}(t)$ is the time-varying air gap during separation (sum of x+y+z), which can be given by: $d_{air}(t) =d_{max}\cdot{sin}^{2}\omega t$	(2d)

Whereas $d_{max}$ is the maximum separation distance and $\omega=2\pi f$ , i.e, the angular frequency and $f$ is the working frequency of TENG.

But since $d_{0}$ is very small compared to $d_{air}(t)$ Equation 2b can be simplified to:

$C_{eff}\left( t \right)=\frac{\varepsilon_{0}A}{d_{air}(t)}$	(2e)

The amount of transferred charge between the two electrodes, as driven by the induced potential, is defined as *Q(t)*, and bound charges +σ and -σ on the positive and negative triboelectric surfaces, respectively. By Gauss’s law, the electric displacement 𝐷 in each layer, ignoring the edge effect, is given by:

i) In air gaps (i = x, y, z):

$D=\varepsilon_{0}E_{air}= \sigma-\frac{Q(t)}{A} \Rightarrow E_{i}= \frac{\sigma-{Q(t)}/A}{\varepsilon_{0}}$	(3a)

ii) In dielectric layers (i = TPU/G and PTFE):

$D=\varepsilon_{0}\varepsilon_{r}E_{i}= \sigma-\frac{Q(t)}{A} \Rightarrow E_{i}= \frac{\sigma-{Q(t)}/A}{\varepsilon_{0}\varepsilon_{r}}$	(3b)

Whereas *Ei* is the electric field across the particular layer. The voltage difference between each layer is *V_i_ =* *E_i_.d_i_.* Thus, the total voltage between the top and bottom electrode layer is given by basic electrodynamics theory and charge conservation:

$V(t)= \sum_{i} E_{i}\cdot d_{i}$	(4a)

Since d1, d2 are very small as compared to $d_{air}(t)$, Equation (4a) can be simplified as:

$V\left( t \right)=E_{TENG} . d_{air}(t)$	(4b)

Whereas, $E_{TENG}$, is the total electrical field generated across the TENG electrodes and *d(t)* is the distance between the electrodes with time.

Substituting all fields across five regions:

$V(t)= E_{x}.x+ E_{TPU/G}.d_{1}+E_{y}.y+ E_{PTFE}.d_{2}+E_{z}.z$	(4c)

Substituting equation (3a), 3(b), and (4a), in equation (4b), we can obtain the V–Q–x relationship for the developed SCVC-TENG.

$V(t)=\left( \frac{\sigma-{Q(t)}/A}{\varepsilon_{0}} \right) \left( d_{air}(t) \right)+\left( \frac{\sigma-{Q(t)}/A}{\varepsilon_{0}\varepsilon_{r1}} \right)d_{1}+\left( \frac{\sigma-{Q(t)}/A}{\varepsilon_{0}\varepsilon_{r2}} \right)d_{2}$	(4d)

$=\left( \sigma-{Q(t)}/A \right) \left[ \frac{d_{air}(t)}{\varepsilon_{0}}+\frac{d_{1}}{\varepsilon_{0}\varepsilon_{r1}}+\frac{d_{2}}{\varepsilon_{0}\varepsilon_{r2}} \right]$	(4e)

In general V-Q-x relationship for TENG is:

$V\left( t \right)=V_{oc}-\frac{Q}{C}$	(5)

Substituting equation (2a) in (4d) gives the V-Q-x relation of SCVC-TENG:

$V(t)=\frac{-Q(t)}{C_{eff}\left( t \right)}+\frac{\sigma A}{C_{eff}\left( t \right)}$	(6)

At an **open-circuit condition,** no charge will flow $\Rightarrow Q=0$.

Thus,

$V_{oc}=\frac{\sigma A}{C_{eff}\left( t \right)}= \sigma\left[ \frac{d_{air}(t)}{\varepsilon_{0}}+\frac{d_{1}}{\varepsilon_{0}\varepsilon_{r1}}+\frac{d_{2}}{\varepsilon_{0}\varepsilon_{r2}} \right]$	(7)

At the highest air gap, d_1_/$\varepsilon_{r1}$ + d_2_/$\varepsilon_{r1}$ is very small compared to $d_{air}\left( t \right)$/$\varepsilon_{0}$, thus, Equation 7 becomes:

$V_{oc}=\sigma\left[ \frac{d_{air}(t)}{\varepsilon_{0}} \right]$	(8)

This voltage derived in **Equation 8** represents the open-circuit voltage generated by the triboelectric capacitor stack (Node 1 to Node 6 from **Figure S1b**). This voltage is further applied across the series combination of CCR 1, CCR 2, and the spark-gap switch shown in **Figure S1b** and is further derived in Section 3 of the manuscript, **Equations 6-8**.

**Figure S5.** Voltage, power, and current density of CCS-TENG.

**Note S2:** **Effect of graphene content on triboelectric performance**

Graphene has been widely used in many smart electronics applications, ranging from super-capacitors to protective shielding on aerospace technologies. (reference) Here, the graphene is utilized to enhance the triboelectric performance of the TPU matrix. The TEM image of the few-layer graphene utilized in this study is displayed in **Figure S8a**. The content of graphene is varied from 1 to 6 wt%, and the resulting composites are named as TPUGX, where X is the content of graphene in wt% (Example: TPUG6 is the TPU/Graphene containing 6 wt % of graphene). For this section of the study, a conventional CS-TENG (CCS-TENG) design was utilized to examine the triboelectric performance in contact-separation mode. The working mechanism of the CCS-TENG design is exhibited in **Figure S6.** Figure **S7a** to **S7g**, displays **t**he empirical waveforms of voltage of all the composites under study with varying frequency. It was observed that TPUG2 showed high voltage output across all frequency of operation. Thus, current and charge output of TPUG2 at 9 Hz of working frequency (highest working frequency) are examined and

**Figure S6.** The working mechanism of the conventional CS-TENG design.

**Figure S7.** Voltage output of control-TENG design with varying frequency of: a) TPU, b) TPUG1, c) TPUG2, d) TPUG3, e) TPUG4, f) TPUG5, g) TPUG6 (the color corresponding to the frequency for **Figure S7a** **to S7g** is given in the inset of **Figure S7a**). h) Current output of the TPUG2 in control-TENG design at 9 Hz frequency. i) Charge output the TPUG2 in control-TENG design at 9 Hz frequency (the color corresponding to the composite for **Figure S7h** **and S7i** is given in the inset of **Figure S7h**).

is displayed in **Figures S7h** **and S7i**. **Figure S8b** summarizes the voltage output and displays the peak-to-peak open-circuit voltage with varying graphene content as well as different frequencies of operation. This figure clearly depicts that the TPU/G composite containing 2 wt% of graphene, i.e., TPUG2, generates high voltage among all the composites across all the frequencies of operation. TPUG2 generated a peak-to-peak V_oc_ of 2.39 kV with a max voltage of 1.6 kV at a high frequency of 9 Hz. At the same frequency, TPUG2 generated a short-circuit current of 84 μA (peak-to-peak) and short-circuit charge of 70 nC, as displayed in **Figure S8c**. A summary of the peak-to-peak voltage, current, and charge of TPUG2 at 9 Hz operation frequency is shown in **Figure S8c**. It depicts that TPUG2 is exhibiting excellent triboelectric properties with the highest V_oc_, current, and charge among all the other composites. In addition, a simulation investigation was carried out to support the observation made from the experimental result using COMSOL Multiphysics to ensure the triboelectric performance of TPUG2. The result of the simulation is in concordance with the experimental result, as shown in **Figure S8d**. In addition, a dielectric spectroscopy of the TPUGX composites is carried out for a frequency range of 10^3^–10^6^ Hz. **Figure S8e** depicts the variation in dielectric constant (ɛ’) with frequency for all the compositions. The dielectric constant increased with the increase in graphene content in the TPU matrix. This can be attributed to the increase in the orientation dipolar polarization due to the addition of graphene.^[16]^ However, the dielectric loss (tan δ) decreased initially till TPUG2, and on further addition of graphene, the loss increased (**Figure**

**Figure S8.** a) TEM image of the few-layer graphene utilized in this study. b) The peak-to-peak open-circuit voltage with varying graphene content and varying frequency of operation. c) Summary of the peak-to-peak voltage, current, and charge of TPUG2 at 9 Hz operation frequency. d) Open circuit voltage - simulation result. e) Dielectric constant with varying graphene content. f) Dielectric loss with varying graphene content.

**S8f**). This initial decrease can be attributed to the suppression of polarization effects caused by hindering the movement of charge carriers since the graphene content is below percolation, leading to a discontinuous filler network inside the TPU matrix.^[17]^ When the concentration of graphene increases, the conductive network formed by the graphene starts to dominate. This conductive network leads to increased conduction losses, causing the dielectric loss to rise significantly. This suggested that TPUG2 exhibited an optimal dielectric constant and a preferable dielectric loss that is essential for a good triboelectric performance. This confirmed the experimental observations, and hence, TPUG2 composite sheet was utilized to fabricate the SCVC-TENG, control-TENG 1, and control-TENG 2, all in combination with PTFE sheet as the opposite triboelectric layer.

**Figure S9.** Contact potential difference (CPD) of a) PTFE, b) TPUG2. C) Difference in tribo-pairs from CPD.

**Figure S10.** CCS-TENG+SG: The empirical waveforms of voltage, current, and charge at varying loads of load resistances of: (a-c) 100 Ω, (d-f) 1 MΩ, (g-i) 47.4 MΩ, and (j-l) 100 MΩ.

**Figure S11.** CCS-TENG+SG+CCRs: The empirical waveforms of voltage, current, and charge at varying loads of load resistances of: (a-c) 100 Ω, (d-f) 1 MΩ, (g-i) 47.4 MΩ, and (j-l) 100 MΩ.

**Figure S12.** SCVC-Floating TPU TENG: The empirical waveforms of voltage, current, and charge at varying loads of load resistances of: (a-c) 100 Ω, (d-f) 1 MΩ, (g-i) 47.4 MΩ, and (j-l) 100 MΩ.

**Figure S13.** SCVC-Floating PTFE TENG: The empirical waveforms of voltage, current, and charge at varying loads of load resistances of: (a-c) 100 Ω, (d-f) 1 MΩ, (g-i) 47.4 MΩ, and (j-l) 100 MΩ.

**Figure S14.** SCVC-TENG: The empirical waveforms of voltage, current, and charge at varying loads of load resistances of: (a-c) 100 Ω, (d-f) 1 MΩ, (g-i) 47.4 MΩ, and (j-l) 100 MΩ.

**Note S3: Effect of Increased Area of Triboelectric Layers and Volumetric Heat Generation Effect**

The empirical waveforms of voltage, current, and charge output of ‘Large SCVC-TENG’ (with triboelectric layers of dimensions of 10×8 cm) are displayed in **Figure S15**. At 100 Ω, it demonstrated a current of 16.5 A, and a charge output of ~ 200 nC. With increase in the external load, the charge output reduced to ~65 nC at 100 MΩ. It followed a similar trend as that of the SCVC-TENG, but with improved performances. It also showed a high-power density of ~11.3 MW at 100 Ω as exhibited in **Figure S15m-n**. This confirmed that the increase in area of the triboelectric layers directly influecnced and improved the power density, voltage, current, and charge output of the SCVC-TENG.

Since this highest area device is capable of generating highest voltage of 6 kV in this study, it is essential to examine the possibility of localized dielectric breakdown and volumetric heat generation under the device's operating field. Initially peak internal field in the TPU/G layer is calculated from the triboelectric surface charge density and the equivalent-circuit relationship developed in Note S1 (modifying Equation 3b):

E_TPU/G_ = σ/(ε_₀_·ε_r_)	(9)

Using the maximum short-circuit transferred charge (200 nC) over the active area of the Large SCVC-TENG (0.008 m²), the surface charge density is σ = 25 µC/m². With the measured dielectric constant of the TPUG2 composite at the device's operating frequency (εr = 14.8 at 4 Hz), the peak internal field is:

ETPU/G = σ/(ε₀·εr) = 0.19 MV/m

This is ~92-fold below the rated dielectric strength of the TPU matrix used in this study (Desmopan 385 E, Covestro Deutschland AG; 17.5 MV/m). The margin is substantially larger and this confirms that the polymeric material, i.e., TPU under study will not undergo dielectric breakdown.

In addition, the volumetric dielectric loss is estimated, using this same field together with the measured dielectric loss factor at the operating frequency (tan δ = 0.75 at 4 Hz), the volumetric heat generation is estimated as:

Pv = 2π·f·ε₀·εr·tan δ·E² = 90 W/m³

which, across the 300 µm thickness of the TPU/G film, corresponds to an areal heat generation rate of 27 mW/m². This is negligible relative to the passive convective dissipation capacity of the device surface, indicating that dielectric loss heating does not pose a meaningful thermal durability concern under the operating conditions demonstrated in this work.

**Figure S15.** Large CCR-TENG: The empirical waveforms of voltage, current, and charge at varying loads of load resistances of: (a-c) 100 Ω, (d-f) 1 MΩ, (g-i) 47.4 MΩ, and (j-l) 100 MΩ. (m) Power density, current and charge comparison at loads of 100 Ω, 1 MΩ, 47.4 MΩ, and 100 MΩ. (n) Zoomed image of Figure S13m (as shown in its inset).

**Note S4: Theoretical Analysis with Field-Dependent Discharge Mechanism for Peak Current and Power**

This section extends the equivalent circuit model of **Figure S1b**, validated in Note S1 and Section 3 of the manuscript for the output voltage, to predict peak current and power under load, and benchmarks these predictions against direct experimental measurement. To model the CCR-TENG-spark gap system, we incorporate field-dependent discharge dynamics, where the spark initiation and current are governed by the electric field (*E*) and plasma physics. Since the charge discharge is caused by an air breakdown across the spark gap (**Figure 2**), the voltage, current, and power output follow a field-dependent discharge mechanism. Based on this mechanism, air breakdown occurs when the electric field across the TENG electrodes, i.e., $E_{TENG}$exceeds the dielectric breakdown strength ($E_{BD}$) of the medium between the spark gap, i.e., air in this study ($E_{BD}$~3 MV/m). Thus, according to this condition and **Equation 6** of the manuscript:

$E_{TENG}= \frac{V(t)}{dt} \geq E_{BD}$	(10)

Since maximum power and current are generated when dt = 2mm, the maximum theoretical voltage output, V(t), is 6 kV.

The key assumptions made in this study are:

Voltage, V(t): 6 kV

Spark gap: 2 mm

$E_{BD}$: 3 MV/m

Plasma conductivity, *σ_p_*: ~10^4^ (for air sparks)

Cross-sectional area of plasma channel in case of all external load, *A_p_*: 2.5 × 10^-8^ m^2^

It is calculated from the measured diameter of the observed plasma channel in this study, as shown in **Figure S16**. The plasma channel is assumed to be cylindrical.

**Figure S16.** Measured diameter of the observed plasma channel.

In a short-circuit condition, the peak current with losses due to the external load resistance can be given by:

$I_{Peak}= \frac{V(t)}{R_{spark}+ R_{load}}$	(11)

Whereas, $R_{load}$, is the external load resistance, and $R_{spark}$, is the resistance of the spark. The spark resistance is time-dependent but can be approximated as:

$R_{spark}= \frac{dt}{\sigma_{P}\cdot A_{P}}$	(12)

Finally, the peak Power can be given by:

$P_{Peak}= V(t)\cdot I_{Peak}$	(13)

**Table S2.** Comparison of theoretical and experimental peak current and power output

| External load resistance, $R_{load}$ (Ω) | Peak current, $I_{Peak}$ (A) | | Peak power, $P_{Peak}$ (W) | |
| --- | --- | --- | --- | --- |
|  | Theoretical | Experimental | Theoretical | Experimental |
| 100 | 55.5 | 14 | 333 k | 19.6 k |
| 1 M | 5.9 m | 4.5 m | 35.4 | 20.25 |
| 47.4 M | 0.126 m | 99.6 μ | 0.75 | 0.47 |
| 100 M | 56 μ | 50 μ | 0.336 | 0.25 |

The comparison of the theoretical and experimental peak current and peak power output is given in **Table S2**. It is also plotted and displayed in **Figure S17**. Even though the current is not varying as much, it is very clear from this comparison that at low external load resistances, the experimental power deviates very much from the theoretical value, as compared to the high external load resistances. This can be explained by introducing a charge transfer efficiency factor (η), which can be given by:

$\eta= \frac{P_{exp}}{P_{theory}}$	(14)

Evidently, at low external load of 100 Ω, the SCVC-TENG exhibited a charge transfer efficiency of only 6 % ($\eta=0.06$). However, at higher loads of 1 MΩ, 47.4 MΩ, and 100 MΩ, it exhibited 57 % ($\eta=057)$, 63 % ($\eta=0.63)$, and 74 % ($\eta=0.74)$, respectively. At low external loads ($R_{load}$<100 Ω), the actual voltage (experimental) is limited by the resistor. Thus, at 2 Ω, when the theoretical voltage was 6 kV, the real experimental voltage was capped at 1070 V. This is the reason for the high deviation in the theoretical and experimental power output at low loads. However, at high external loads ($R_{load}\geq$1 MΩ), voltage does not get influenced by resistors, but is limited by the plasma losses, such as losses in spark formation, as ionization energy, field inhomogeneities, such as humidity and roughness on the surface of electrodes.

**Figure S17.** Theoretical and experimental output comparison of a) Peak current, and b) Peak power.

**Note S5: Working mechanism of the spark switch**

A custom-made spark switch was developed in this study, as shown in **Figure S18a**. It consists of two holders for holding the two plates of the spark switch and a control knob for controlling the gap of the spark switch. By rotating the knob, the spark gap can be increased or decreased to control the turn-on voltage as needed. **Figure S18b** displays the observed spark across the spark gap.

**Figure S18.** a) Spark switch. b) Spark generated across the spark gap.

**Note S6: Energy efficiency of SCVC-TENG**

This energy transform efficiency from mechanical to electrical power is calculated using the equation^[12]^:

$\eta\approx\frac{W_{\text{elec}}}{W_{\text{mechanical}}}=\frac{S\sigma^{2}}{2\varepsilon_{0}F}$	(15)

Where, *W_elect_* and *W_mechanical_* are the electrical output from the TENG device and the mechanical work put onto the TENG, respectively, and S is the overlapping surface area, σ is the surface charge density, F is the force applied.

**Figure S19**. Voltage output of SCVC-TENG with varying humidity from 45% to 75% relative humidity.

**Figure S20.** a) Schematic illustration of the circuit for voltage and charge measurements after stage 1 of the EM system. b) Voltage and charge output at 0.5 mm spark gap. c) Voltage and charge output at 2 mm spark gap. d) Cyclic stability of voltage output after stage 1 of the EM system when examined for 1 hour.

**Figure S21.** Voltage output of CCS-TENG with the 1^st^ stage of EM.

**Note S7: Power transfer efficiency of large SCVC-TENG while lighting 221 W rated LED combination.**

To calculate power transfer efficiency, we compare the energy delivered to the bulb load during a single discharge event against the energy available in the charge reservoir system at the moment of spark-gap breakdown. Thus, the power transfer efficiency is given by η = E_delivered_ / E_available_. **Figure S22a** show the V-Q plot of the large SCVC-TENG to give the energy available for discharge. The obtained energy available for discharge is 128.7 µJ. **Figure S22b** exhibits the V-Q plot obtained across the lamp load (19.7 MΩ) during a single discharge event to give the energy delivered to the LED lights during the lighting, i.e., 76.9 µJ. This gives a power transfer efficiency, η of 59.7 %.

**Figure S22.** The V-Q plots of the large SCVC-TENG for: a) energy available for the LED lights. b) energy delivered to the LED lights during lighting.

**Figure S23**. Observed voltage when powering a) the green LED continually. b) Hygrothermometer continually.

**Figure S24.** Schematic illustration and Top view of the: a) SCVC-TENG, b) control-TENG, c) SCVC-TPU floating TENG, and d) SCVC-PTFE floating TENG.

**References**

1. Z. Wang, W. Liu, W. He, et al., “Ultrahigh Electricity Generation from Low-Frequency Mechanical Energy by Efficient Energy Management,” *Joule* *5*, no. 2 (2021): 441–455, https://doi.org/10.1016/j.joule.2020.12.023.

2. H. Wu, S. Wang, Z. Wang, and Y. Zi, “Achieving Ultrahigh Instantaneous Power Density of 10 MW/M2 by Leveraging the Opposite-Charge-Enhanced Transistor-like Triboelectric Nanogenerator (OCT-TENG),” *Nature Communications* *12*, no. 1 (2021): 5470, https://doi.org/10.1038/s41467-021-25753-7.

3. G. Cheng, Z.-H. Lin, L. Lin, Z. Du, and Z. L. Wang, “Pulsed Nanogenerator with Huge Instantaneous Output Power Density,” *ACS Nano* *7*, no. 8 (2013): 7383–7391, https://doi.org/10.1021/nn403151t.

4. Z. Wang, Q. Tang, C. Shan, et al., “Giant Performance Improvement of Triboelectric Nanogenerator Systems Achieved by Matched Inductor Design,” *Energy & Environmental Science* *14*, no. 12 (2021): 6627–6637, https://doi.org/10.1039/D1EE02852A.

5. Y. Fu, and H. Ruan, “Advanced Modular Rotary Triboelectric Nanogenerator: Pushing Boundaries in Peak Power Density and Impedance Reduction,” *Nano Energy* *134* (2025): 110557, https://doi.org/10.1016/j.nanoen.2024.110557.

6. T. Zhao, B. Niu, B. Liu, et al., “A Rotating Tower-like Triboelectric Nanogenerator for Ultrahigh Charge Density Breakthrough,” *Nano Energy* *108* (2023): 108204, https://doi.org/10.1016/j.nanoen.2023.108204.

7. S. Li, Z. Zhang, F. Yang, et al., “Transistor-like Triboiontronics with Record-High Charge Density for Self-Powered Sensors and Neurologic Analogs,” *Device* *2*, no. 6 (2024): 100332, https://doi.org/10.1016/j.device.2024.100332.

8. G. Jian, Q. Meng, N. Yang, et al., “Superhigh Charge Density and Direct-Current Output in Triboelectric Nanogenerators via Peak Shifting Modified Charge Pumping,” *Nano Energy* *102* (2022): 107637, https://doi.org/10.1016/j.nanoen.2022.107637.

9. Z. Li, C. Yang, Q. Zhang, et al., “Standardized Volume Power Density Boost in Frequency-Up Converted Contact-Separation Mode Triboelectric Nanogenerators,” *Research* *6* (2023): 0237, https://doi.org/10.34133/research.0237.

10. M.-H. Jang, S. P. Rabbitte, A. Frendi, R. T. Conners, Y. Lei, and G. Wang, “Wide Bandwidth High-Power Triboelectric Energy Harvesting by Scotch Tape,” *ACS Omega* *10*, no. 3 (2025): 2778–2789, https://doi.org/10.1021/acsomega.4c08590.

11. J. Chun, B. U. Ye, J. W. Lee, et al., “Boosted Output Performance of Triboelectric Nanogenerator via Electric Double Layer Effect,” *Nature Communications* *7*, no. 1 (2016): 12985, https://doi.org/10.1038/ncomms12985.

12. Y. H. Liufu, C. K. Tse, G. Yang, et al., “Achieving Ultrahigh Efficiency of Triboelectric Nanogenerator Energy Harvesting Systems via Hybrid Electronic-Spark Power Management,” *Advanced Functional Materials* *35*, no. 10 (2025): 2415338, https://doi.org/10.1002/adfm.202415338.

13. S. Niu, and Z. L. Wang, “Theoretical Systems of Triboelectric Nanogenerators,” *Nano Energy* *14* (2015): 161–192, https://doi.org/10.1016/j.nanoen.2014.11.034.

14. S. Niu, Y. Liu, X. Chen, et al., “Theory of Freestanding Triboelectric-Layer-Based Nanogenerators,” *Nano Energy* *12* (2015): 760–774, https://doi.org/10.1016/j.nanoen.2015.01.013.

15. S. Niu, Y. Liu, S. Wang, et al., “Theoretical Investigation and Structural Optimization of Single‐Electrode Triboelectric Nanogenerators,” *Advanced Functional Materials* *24*, no. 22 (2014): 3332–3340, https://doi.org/10.1002/adfm.201303799.

16. S. Sharma, A. Thapa, S. Pramanik, C. Sengupta, and T. Mondal, “Graphene‐Infused Sustainable Rubber‐Based Triboelectric Nanogenerator For Real‐Time Human Motion Monitoring,” *Small* *20*, no. 46 (2024): 2404771, https://doi.org/10.1002/smll.202404771.

17. C.-Y. Lee, and C.-W. Chang, “Dielectric Constant Enhancement with Low Dielectric Loss Growth in Graphene Oxide/Mica/Polypropylene Composites,” *Journal of Composites Science* *5*, no. 2 (2021): 52, https://doi.org/10.3390/jcs5020052.
